# Ge_5_ Clusters in the Trivalent Rare-Earth Compound Sm_3_Ge_5_

**DOI:** 10.1021/acs.inorgchem.5c02417

**Published:** 2025-09-13

**Authors:** Julia-Maria Hübner, Riccardo Freccero, Wilder Carrillo-Cabrera, Marcus Schmidt, Walter Schnelle, Ulrich Schwarz

**Affiliations:** † Faculty of Chemistry and Food Chemistry, 9169 TUD Dresden University of Technology, Dresden 01062, Germany; ‡ Dipartimento di Chimica e Chimica Industriale, Università degli Studi di Genova, Via Dodecaneso 31, Genova I-16146, Italy; § Max Planck Institute for Chemical Physics of Solids, Dresden 01187, Germany

## Abstract

The compound Sm_3_Ge_5_ adopts two modifications with Pearson symbols *hP*16 (AlB_2_-derivative) and *oF*64 (defect α-ThSi2-type) upon synthesis at ambient pressure. Synthesis at extreme conditions grants access to the modification *oS*32 (Pu_3_Pd_5_-type). High-pressure high-temperature treatment of prereacted element mixtures yields Pu_3_Pd_5_-type Sm_3_Ge_5_, space group *Cmcm* with lattice parameters *a* = 9.42813(9), *b* = 7.56296(7), and *c* = 9.67056(8) Å. The atomic arrangement refined from powder X-ray diffraction data is confirmed by transmission electron microscopy measurements. The crystal structure features Ge_5_ square pyramidal units. The topology of the Electron Localizability Indicator (ELI-D) supports the formation of a bicyclo[1.1.1]­pentagermanide cluster composed of two- and three-bonded Ge species, resulting in an electron balance comprising excess electrons. The bonding analysis in position space further reveals the presence of polar covalent interactions between both germanium and the rare-earth metal and among the Ge atoms constituting the base of the Ge_5_ pyramidal units, pointing to a complex bonding scenario that is difficult to rationalize by electron counting rules. Sm_3_Ge_5_ shows a metallic conductivity. Heat capacity and magnetization measurements indicate a 4*f* ^5^ electron configuration and thus the trivalent state of the Sm ions. The magnetic moments of Sm in Sm_3_Ge_5_ order antiferromagnetically at 20.4 K.

## Graphical abstract

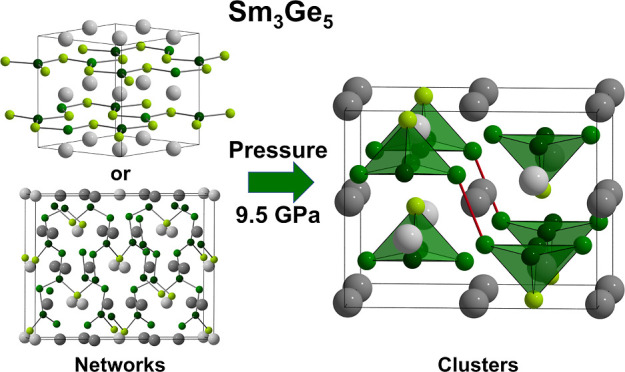

## 1 Introduction

Compounds of alkaline earth or rare-earth metals and group 14 elements exhibit a wide panoply of crystal structures ranging from close-packed arrangements to host–guest frameworks. Although the electronegativity difference of the elements would suggest a complete electron transfer, thus implying the formation of Zintl anions, the electron count does not follow simple rules in many cases. The special bonding situations give rise to additional degrees of freedom in composition and crystal structure and, therefore, physical properties.

Binary alkaline-earth and rare-earth metal (*M*) tetrels (*Tt*, here Si, Ge, Sn, and Pb) with the general formula *M*_3_ *Tt*_5_ pose an interesting area of research, as the Si and Ge analogues predominantly crystallize in defect variants of the AlB_2_- or α-ThSi_2_-type structure pointing at a large variety of different structural and magnetic ordering scenarios.^1–35^ The same holds for the binary Sm–Ge system,^36^ in which the two polymorphs of Sm_3_Ge_5_, *oF*64, and *h* *P*16 crystallize in such ordered defect variants *M*_3_ *Tt*_5_.^20^ Here, the metal atoms adopt the oxidation state +3. Additionally, for divalent metals like Ba_3_Ge_5_ ^5^ and Eu_3_Ge_5_ ^21^ or mixed-valent +2/+3 Yb_3_Ge_5_ and the respective compounds comprising the heavier tetrel elements (Sn, Pb), defect-free Pu_3_Pd_5_-type structures comprising five-atomic germanium clusters are conveyed.^3–5,13,16,34,35^

Here, we report on the high-pressure, high-temperature synthesis of a new Sm_3_Ge_5_ phase with Sm in the oxidation state +3, as evidenced by magnetic property measurements. The chemical bonding scenario is investigated in detail by quantum chemical methods in direct space.

## 2 Experimental Section

### 2.1 Synthesis

Sample handling, except for high-pressure synthesis itself, was performed in argon-filled glove boxes (MBraun, H_2_O and O_2_ < 0.1 ppm). The precursor mixture was prepared by arc melting of samarium (Lamprecht, 99.9%) and germanium (Chempur, 99.9999+%) with an optimized excess of 9% Sm (based on mass loss) to compensate evaporation loss. The resulting material was thoroughly ground and put into a BN crucible before being placed in a MgO octahedron (edge length 18 mm). High-pressure high-temperature synthesis was performed in a multianvil Walker-type module^37^ at 9.5 GPa and temperatures between 1420 and 1570 K for 30 to 300 min (with an error both for pressure and temperature of ±10%) as longer annealing times proved beneficial for product yield. Additionally, a treatment at 820 K for 7–50 h before quenching or cooling within 5–20 h under load was tested to facilitate crystallization. However, no improvement of the crystal quality was found, and, therefore, no specimen suitable for single-crystal X-ray diffraction was isolated. Synthesis at ≤7 GPa did not yield the target phase, and above 11 GPa, hitherto unidentified side phases were obtained (for further details on the results of the respective experiments, see the Supporting Information, Table S1). Calibration of pressure and temperature by resistance changes of bismuth and thermocouple-calibrated runs were realized prior to the experiments. No uncommon hazards were noted.

### 2.2 Powder X-ray Diffraction

Phase assignment, determination of unit cell parameters, and Rietveld refinements were conducted on the basis of powder X-ray diffraction (PXRD) data measured with a Guinier system (Cu *Kα*_1_ radiation, *λ* = 1.540598 Å, graphite monochromator, Huber 670 camera, 5° ≤ 2*θ* ≤ 100°, Δ2*θ* = 0.005°) at room temperature. Rietveld refinements were conducted with Jana2020.^38^

### 2.3 Scanning Electron Microscopy

Prior to analysis, samples were fixed on a carbon pad settled on an aluminum sample holder or were embedded in paraffin and polished with a suspension of diamond powders (grain sizes 6, 3, and 0.25 μm). Scanning electron microscopy (SEM) (acceleration voltage *U*_acc_ = 5 kV) was performed using a SU8020 electron microscope (Hitachi) equipped with a multidetector system for secondary and low-energy backscattered electrons and an Oxford Silicon Drift Detector (SDD) X-MaxN for semiquantitative energy-dispersive X-ray (EDX) spectroscopy (*U*_acc_ = 20 kV).^39^

### 2.4 Transmission Electron Microscopy

Selected area electron diffraction (SAED) was used for crystal structure characterization. Specimens suitable for the TEM investigation were prepared by grinding a piece of sample in an agate mortar. Diffraction experiments were performed on a FEI Tecnai F30-G2 supertwin microscope operating at 300 kV. The microscope was equipped with a CCD camera (GATAN Inc.) and a standard double-tilt holder (GATAN Inc.) with a tilting range of ±46° of the holder axis and ±30° perpendicular to it. The powdered particles were deposited on a holey carbon film supported on a copper TEM grid. Several particles oriented in different directions were used for the SAED electron diffraction study.

### 2.5 Thermal Analysis

Differential scanning calorimetry analysis was performed with a NETZSCH DSC 404C device (NETZSCH, Selb, Germany) using a corundum crucible with a lid and heating rates of 10 K min^–1^ under an argon atmosphere.

### 2.6 Physical Properties

A pellet (about 6 mm diameter) cold pressed from polycrystalline powder was affixed to a puck using GE-varnish (IMI 7031) and contacted with two Pt wires (25 μm diameter, GoodFellow) using Ag-filled epoxy (Plano GmbH). The electrical resistance (DC resistivity probe) was measured in a temperature range from 5 to 300 K in a cryogen-free measurement system (CFMS, Cryogenic Ltd.). Using the same pressed material, magnetization was determined with an MPMS3 magnetometer (Quantum Design). After zero-field cooling, magnetization was measured in temperature sweeps 1.9 → 400 → 1.9 K (*zfc* and *fc*). Magnetization isotherms (0 T → + 7 T → −7 T → + 7 T) were taken at selected temperatures between 12 and 200 K after cooling the sample in zero field from 200 K. Heat capacity in zero (1.8 → 300 K) and applied magnetic fields of 1.5, 3, 6, and 9 T (1.8 K → 30 K) were measured with the HC option of a Quantum Design PPMS. The sample was affixed with a weighted amount of Apiezon N grease.

### 2.7 Computational Details

The density functional theory-based all-electron Full-Potential Local-Orbital (FPLO)^40,41^ code was employed to perform quantum chemical calculations selecting the Perdew–Burke–Ernzerhof (PBE) exchange–correlation functional.^42^ Spin-polarized calculations were conducted for Sm_3_Ge_5_ assuming both ferromagnetic (FM) and antiferromagnetic (AFM) ordering. Given the different multiplicity of the Sm Wyckoff sites in the *Cmcm* space group, i.e., 4*c* and 8*e*, the symmetry was reduced to the orthorhombic *Pmma* space group (No. 51) to ensure the same number of spin-up and spin-down samarium atoms per cell (crystallographic data are reported in Table S6). A 16 × 20 × 16 and 16 × 16 × 20 *k*-mesh was employed to sample the Brillouin zone for the *Cmcm* (FM) and the *Pmma* (AFM) structures, respectively. All atomic positions were optimized, and a scalar-relativistic treatment was used to approximate the relativistic effects. As calculations with different on-site Coulomb repulsion parameters *U* led to convergence issues, also using the typical value of 8 eV in the FPLO method,^43,44^ the 4*f* valence states of the samarium species were moved into the core, setting an occupation of five, as indicated by physical property measurements (4*f* ^5^), and the remaining *f*-polarization orbitals (POs) were removed from the valence sector. This enables us to avoid wrong matrix elements between the core-4*f* and the *f*-valence POs. Population analysis for each atom showed the net never exceeding the gross population.

Aiming to perform a chemical bonding analysis in the position space, the electron density (ED) and the electron localizability indicator (ELI-D)^45,46^ scalar fields were calculated on an equidistant grid of ∼0.05 Bohr, thanks to a module implemented within the FPLO code.^47^ For this purpose, the *k* mesh was reduced to 8 × 8 × 10. The topologies of calculated ED and ELI-D were analyzed by means of the DGrid program,^48^ applying the mathematical approach of the Bader’s Quantum Theory of Atoms In Molecules (QTAIM).^49^ Following this procedure, the crystal space is partitioned into nonoverlapping, space-filling regions, referred to as QTAIM (*X*) and ELI-D (*B*_i_) basins, bounded by surfaces of zero-flux in ED and ELI-D gradient, respectively. Integration of ED within QTAIM basins yields their average atomic populations *N̅*(*X*), from which effective atomic charges *Q* ^eff^(*X*) are derived; integration of the ED within ELI-D basins provides the electronic populations of core and valence basins $\mathrm{N̅}\left(B_{i}\right)$ . Once these two distinct spatial partitioning are obtained, DGrid enables the ELI-D/QTAIM basins intersection,^50–52^ thereby allowing the evaluation of ELI-D basins’ atomicity,^53^ defined as the number of QTAIM atoms intersecting a given ELI-D valence basin, and enabling the determination of the contributions of individual QTAIM atoms *X* to the bond populations of ELI-D valence basins $\mathrm{N̅}\left(B_{i}^{X}\right)$ and then of bond polarities, quantified by means of the bond fractions $p\left(B_{i}^{X}\right)=\frac{\mathrm{N̅}\left(B_{i}^{X}\right)}{\mathrm{N̅}\left(B_{i}\right)}$ .^51,52,54^ Thus, nonpolar bonds and lone pairs are indicated by bond fractions of 0.5 and 1.0, respectively, whereas polar bonds get values intermediate between 0.5 and 1.0. In this way, references to known electronegativity scales are not required. In order to gain an in-depth understanding of the bonding in Sm_3_Ge_5_, a model compound was chosen, namely, La_3_Ge_5_. Given the trivalent nature of samarium, it was obtained by replacing Sm with La in the computationally optimized structure of Sm_3_Ge_5_. This choice was made necessary due to issues encountered in the calculation of the ELI-D for the title compound (see also the Results and Discussion paragraph).

To validate this methodology and ensure that the peculiarities observed in the bonding were not attributable to the chosen computational approach, additional calculations of both ED and ELI-D have been performed for La_3_Sn_5_ (see Section 8 in the Supporting Information), using crystallographic data published by Klem et al.,^4^ and for the hypothetical La_3_Ge_5_ compound obtained by full crystal structure optimization (details in Section 9 of the Supporting Information). The ParaView application^55^ together with a dedicated plug-in^56^ enabled the visualization of the calculated scalar fields and their basins.

## 3 Results and Discussion

High-pressure, high-temperature reactions aiming at the synthesis of SmGe_3_ ^57^ yield a unknown second phase with composition Sm_38.0(5)_Ge_62.0(5)_, as determined by energy-dispersive X-ray spectroscopy analyses. The average composition of this byproduct is compatible with a 3:5 ratio. Subsequent targeted synthesis leads to samples containing Sm_3_Ge_5_ as the majority phase, but the samples still contain either ∼2 wt % Sm_5_Ge_3_ and 4 wt % SmGe_3_ (Figure 1) or less than approximately 5% of a secondary product (see Table S1), which remained unidentified. Despite SEM/EDXS and TEM analysis, the characterization is hampered by grain intergrowth and a small domain size (Figure S1, Table S2).

**Figure 1:**
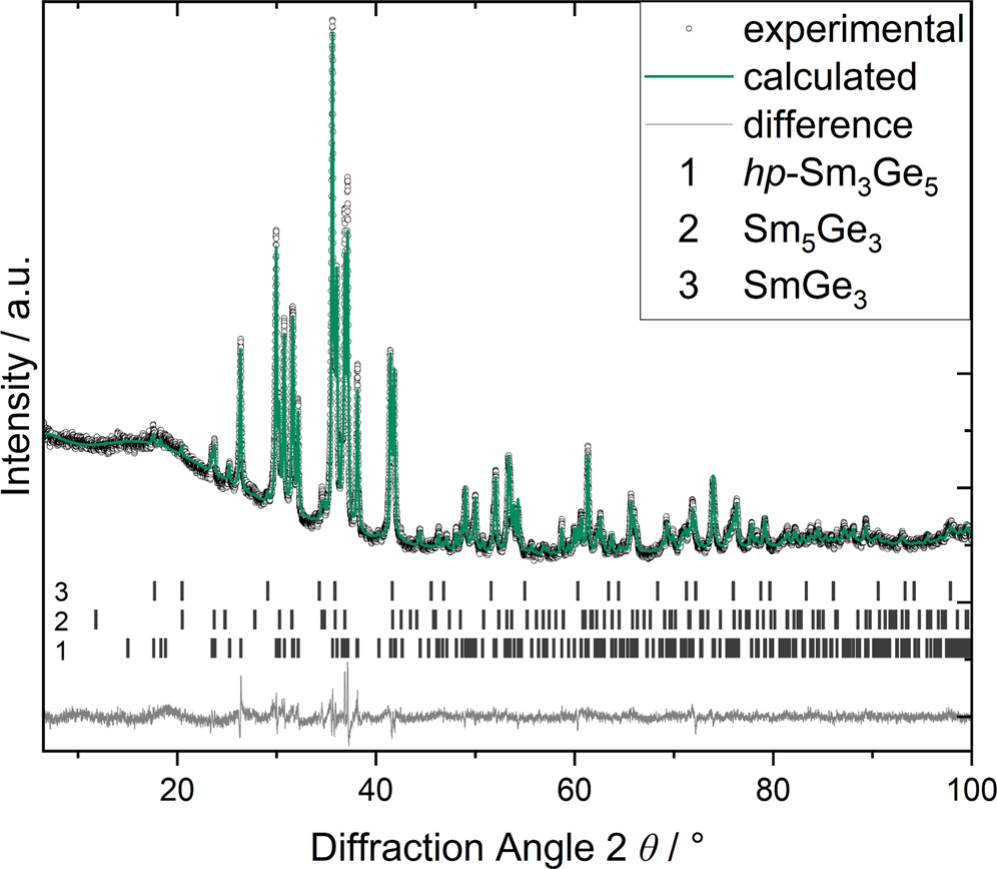
Powder XRD pattern (Cu *Kα*_1_ radiation) of Sm_3_Ge_5_ and results of Rietveld refinement. The sample contains approximately 2 wt % Sm_5_Ge_3_ and 4 wt % SmGe_3_.

The X-ray powder diffraction pattern (Figure 1) denotes that the high-pressure form of Sm_3_Ge_5_ crystallizes in a Pu_3_Pd_5_-type^58^ structure with lattice parameters *a* = 9.42813(9), *b* = 7.56296(7), and *c* = 9.67056(8) Å. Neither for the target compound nor the side product, crystals suitable for single-crystal X-ray diffraction could be isolated from the silver-colored ingots.

For substantiation of the space group symmetry, several selected area electron diffraction patterns were collected on thin, single-domain lamellar samples (Figure 2). Taking into account multibeam effects, the observed reflection conditions correspond to *hkl*: *h* + *k* = 2*n*; 0*kl*: *k* = 2*n*; *h*0*l: h* = 2*n*, *l* = 2*n*; *hk*0: *h* + *k* = 2*n* and *h*00: *h* = 2*n*; 0*k*0: *k* = 2*n*; 00*l*: *l* = 2*n*, confirming that the high-pressure phase Sm_3_Ge_5_ crystallizes in space group *Cmcm* (No. 63).

**Figure 2:**
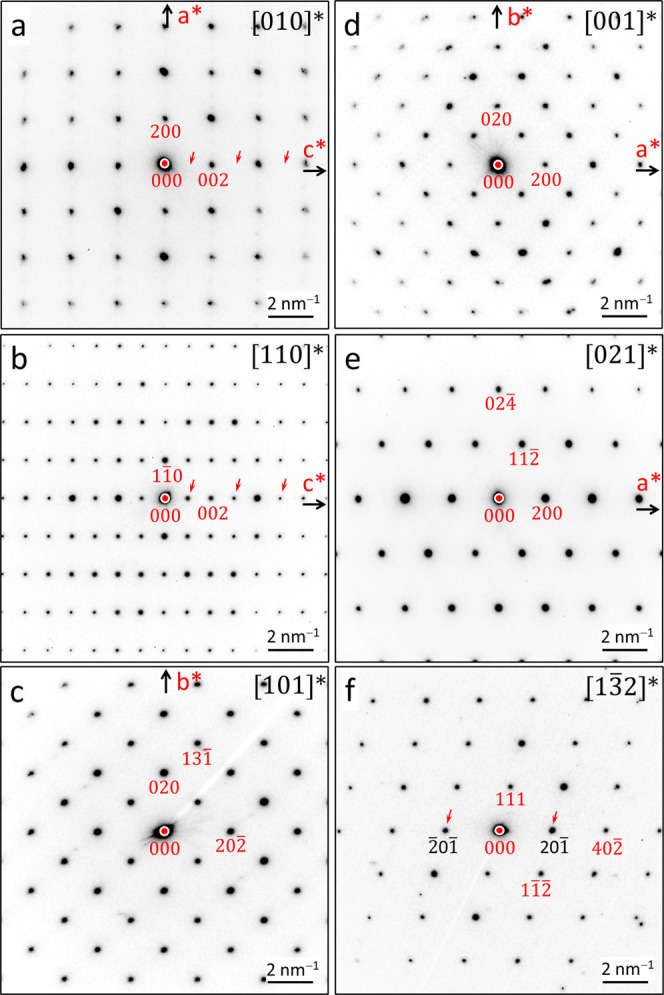
SAED images obtained from individual crystallites of a Sm_3_Ge_5_ powder along the zone directions (a) [010]*, (b) [110]*, (c) [101]*, (d) [001]*, (e) [021]*, and (f) [13̅2]*. Taking into account multibeam dynamical interactions on thick TEM lamellas for (b,f), the observed reflections conditions are *hkl*: *h* + *k* = 2*n*; *h*0*l*: *h* = 2*n*, *l* = 2*n*; 0*kl*: *k* = 2*n*; *hk*0: *h* + *k* = 2*n* and *h*00: *h* = 2*n*; 0*k*0: *k* = 2*n*; 00*l*: *l* = 2*n*; indicating that the phase Sm_3_Ge_5_ crystallizes in space group *Cmcm* (No. 63). The observation of some significant intensities, which seemingly violate the systematic reflection conditions, is restricted to certain orientations and, thus, attributed to multiple scattering (see red arrows).

Rietveld refinements result in residuals *R*_P_ = 0.0371, *wR*_P_ = 0.0372 (Figure 1, Tables 1 and S3). The refined atomic coordinates (Table S3) essentially correspond to those obtained from quantum chemical optimization (Table S7).

**Table 1:** Data Collection (293 K), Crystal Structure Refinement, and Crystallographic Information for Sm_3_Ge_5_

composition	Sm_3_Ge_5_
space group, Pearson symbol, structure type	*Cmcm* (no. 63), *oS*32, Pu_3_Pd_5_
lattice parameters	
*a*/Å	9.42813(9)
*b*/Å	7.56296(7)
*c*/Å	9.67056 (8)
*V*/Å^3^	689.55(1)
formula units, *Z*	4
density/g cm^–3^	7.84
formula weight	814
source	Cu *K*α_1_ radiation, *λ* = 1.54175 Å
measurement range	6.5 ≤ 2*θ* ≤ 100.4°
measd points/reflns.	19481/215
*R*(P)/w*R*(P)/GOF	0.0371/0.0372/1.10

The crystal structure can be described as an anionic substructure consisting of Ge_5_ square pyramidal clusters separated by samarium atoms (Figure 3). Sm1 is coordinated by 9 Ge and 4 Sm atoms and Sm2 by 12 Ge and 5 Sm atoms (for interatomic distances, see Table S4). Under consideration of the ambient pressure modifications^20^ featuring two- or three-dimensional anionic partial structures, respectively, the finding of isolated polyanionic units in the high-pressure phase Sm_3_Ge_5_ appears to be counterintuitive. However, the average volume per atom is smaller for (*oS*32)­Sm_3_Ge_5_ than for the ambient pressure modifications so that the formation of this atomic arrangement upon compression is in accordance with Le Chatelier’s principle.

**Figure 3:**
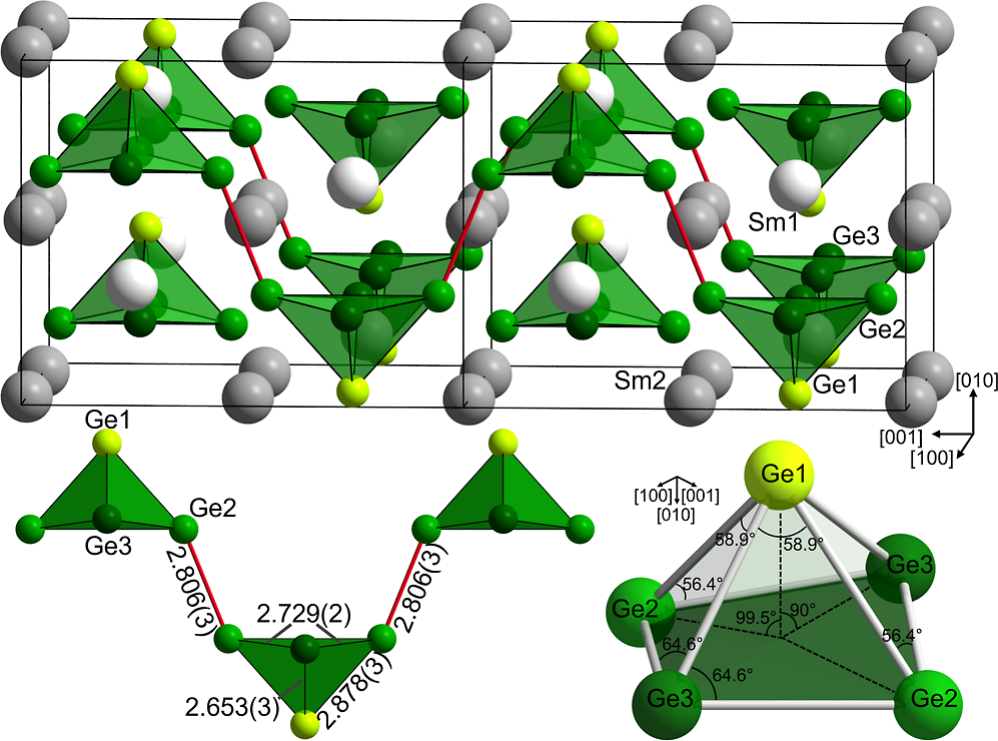
(Top) Crystal structure of Sm_3_Ge_5_ with anionic Ge_5_ clusters depicted in green. The position of the unit cell is indicated by black lines. (bottom, left) Polymeric chain of square pyramidal clusters with interatomic distances given in Å. (bottom, right) Bond angles in the cluster units of Sm_3_Ge_5_.

The crystal structure of Sm_3_Ge_5_ may be described as defect variety of SmGe_3_,^57^ which crystallizes in a superstructure variant of the Cu_3_Au-type. Both atomic arrangements contain arrays of corner-sharing polyhedra. In the case of SmGe_3_, octahedral [Ge_6_]-units are condensed by shared vertices. In Sm_3_Ge_5_, square pyramids alternate with trigonal bipyramids, and the units are connected by sharing vertices. The square pyramids may be complemented to octahedral units by an additional Sm atom (Figure 4).

**Figure 4:**
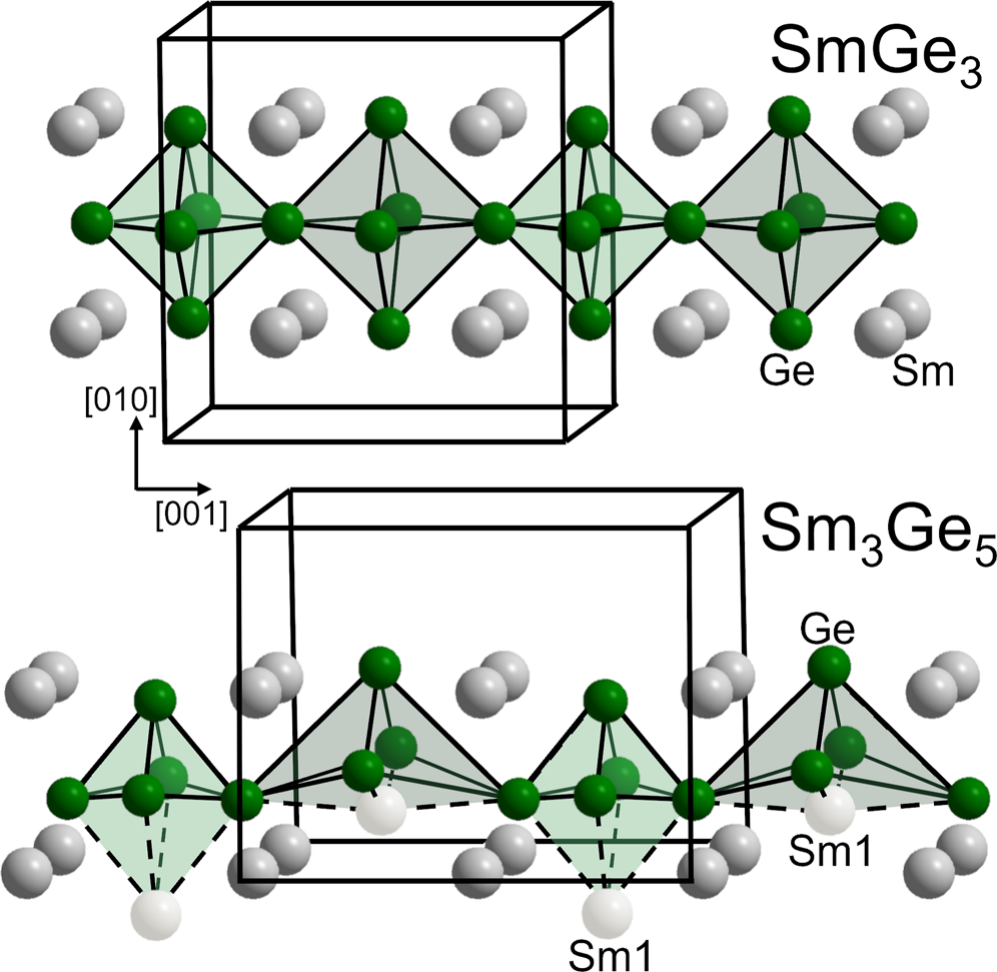
Comparison of the crystal structures of SmGe_3_ and Sm_3_Ge_5_.

The distances in the Ge_5_ square pyramidal clusters range from 2.653(3) to 2.878(3) Å (Figure 3 bottom left, Table S4), being significantly longer than distances observed in elemental Ge (2.45 Å^59^). However, they fall well into the range of other *M*Ge_2–*x*_ compounds.^60^ The comparison with other binary Pu_3_Pd_5_-type tetrel compounds (Table S5) reveals that the interpyramidal distances correlate (linearly) with the ionic radius of the metal (Figure S2).

The square pyramids are slightly distorted (Figure 3, bottom right). The ratio between the exohedral distances in between the square pyramids and the endohedral distances (Figure 3) is similar to the one observed in La_3_Sn_5_ ^4^ but clearly larger than the ones in Pu_3_Pd_5_-type compounds of divalent metals (Figure 5, Table S5).

**Figure 5:**
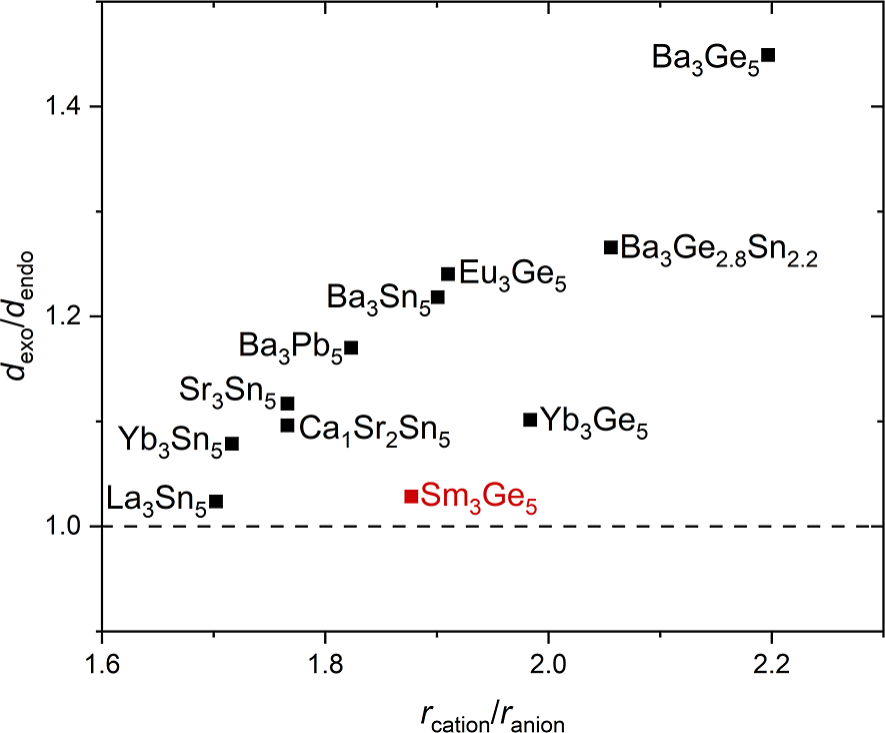
Distances between the square pyramids (exohedral) scaled to the average endohedral distance of selected Pu_3_Pd_5_-type compounds.

Applying established electron rules to initially assess the chemical bonding of Sm_3_Ge_5_ is not trivial, given the possibility of multiple scenarios depending on the number of electrons formally transferred from the rare-earth metal to germanium. In fact, while electroneutrality in *RE*_3_In_5_ phases (*RE* = rare-earth metal), which are isostructural to Sm_3_Ge_5_, is respected by the (*RE* ^3+^)_3_(In_5_^9–^) formula, comprising a *nido*-deltahedral indium cluster,^61^ the same cannot be easily applied when tetrel elements like germanium and tin are involved. Moreover, the chemical bonding in those compounds was described using different scenarios (Table 2).

**Table 2:** Cluster Descriptions of Compounds *M*_3_ *Tt*_5_ (*M* = Sr, Ba, Eu, Yb, La; *Tt* = Ge, Sn)

compound	oxidation state of M	cluster type	electron balance	ref
Sr_3_Sn_5_	+2	arachno Sn_5_^6‑^	(Sr^2+^)_3_(Sn_5_^6–^)	^3^
Ba_3_Sn_5_	+2	arachno Sn_5_^6‑^	(Ba^2+^)_3_(Sn_5_^6–^)	^3^
Sr_3_Sn_5_	+2	nido Sn_5_^4‑^	(Sr^2+^)_3_(Sn_5_^4–^) × 2e^–^	^4^
Eu_3_Ge_5_	+2	[1.1.1] barrelane	(Eu^2+^)_3_(Ge_5_^6–^)	^21^
Yb_3_Ge_5_	+2 (0.6); +3 (0.4)	nido Ge_5_^4‑^	(Yb^2.4+^)_3_(Ge_5_^4–^) × 3.2e^–^	^34^
La_3_Sn_5_	+3	nido Sn_5_^4‑^	(La^3+^)_3_(Sn_5_^4–^) × 5 e^–^	^4^

Here, it is worth noting that for La_3_Sn_5_,^4^ which has the same number of valence electrons as the title compound, the homoatomic Sn–Sn interactions were found to be subsidiary to the heteroatomic Sn–La ones. The interest shown over the years in understanding the chemical interactions occurring in these phases, together with the fact that Sm_3_Ge_5_ is the first germanium representative with a purely trivalent lanthanide metal (see Table S5), has motivated a detailed study of its electronic structure and bonding.

Spin-polarized DFT calculations yield spin magnetic moments for (Sm1, Sm2) of (5.36, 5.38)*μ*_B_ in the ferromagnetic state and of ± (5.41, 5.42)*μ*_B_ for the antiferromagnetic structure, which turns out to be more stable by 1.8 meV/atom. Interestingly, although the approximation of treating the *f*-orbitals as core may lead to unreliable total energies, the energy difference between the FM and AFM structures is in agreement with experimental data. Focusing on the AFM phase, the spin moments result from *s*, *p*, *d*, and *f* contributions of 0.03, 0.03, 0.35­(Sm1)/0.36­(Sm2), and 5.00 *μ*_B_, respectively. The induced spin moments on the Ge atoms are negligible (<0.01 *μ*_B_). It should be kept in mind that given the in-core treatment of the 4*f* orbitals, these values should be regarded as estimates that may require further verification, both experimental and computational. The electronic density of states for the AFM structure is reported in Figure 6 (top) and shows interesting analogies with that of rare-earth monogermanides.^60,62,63^

**Figure 6:**
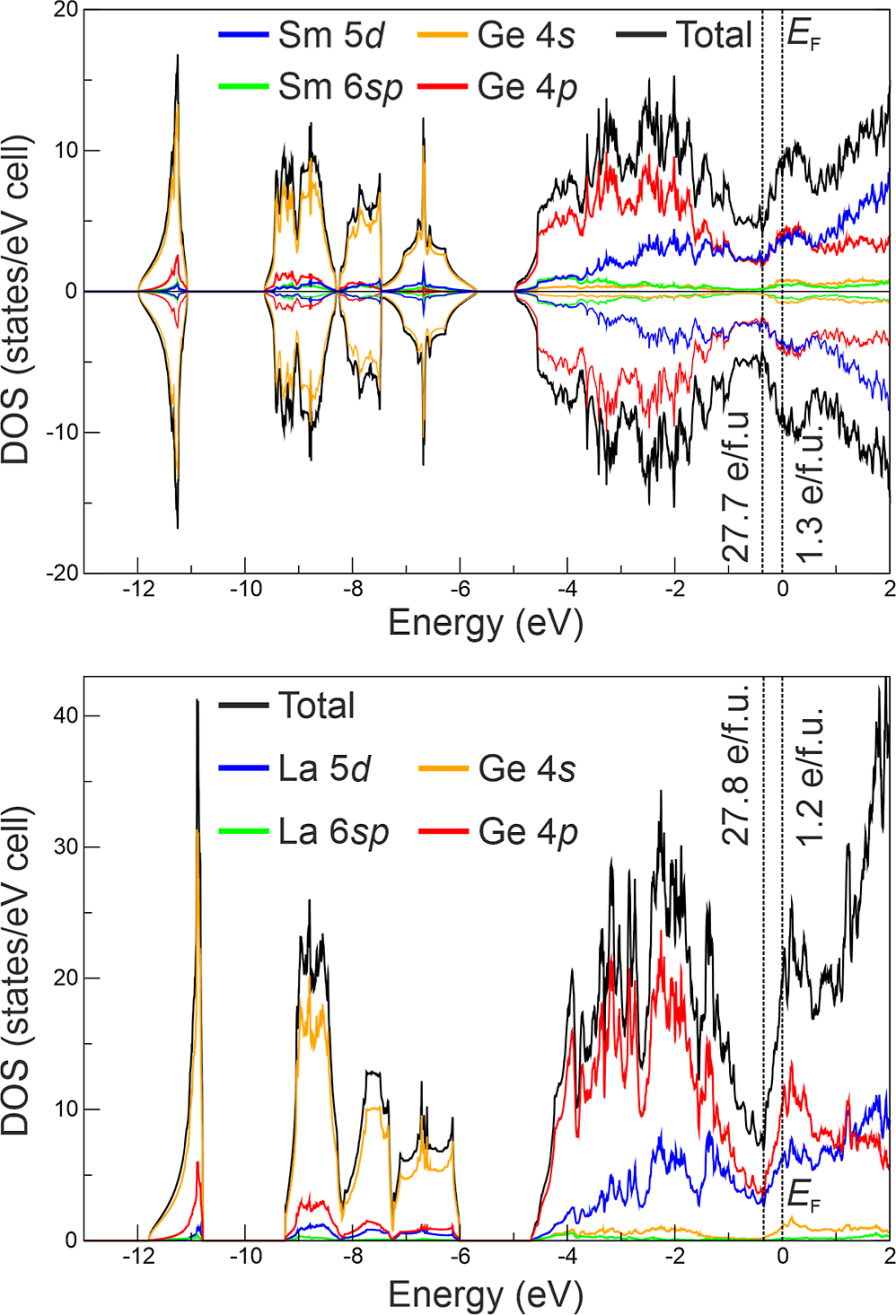
Total and orbital-projected electronic density of states for Sm_3_Ge_5_ (up) with AFM order and La_3_Ge_5_ (bottom). For Sm_3_Ge_5_, both spin channels are displayed.

The DOS region below −5 eV is dominated by Ge 4*s* states, whereas the 4*p* mainly contributes to the range from approximately −5 to 0 eV; they overlap with Sm 5*d* states, with gradually increasing contribution closer toward *E*_F_. This suggests incomplete samarium ionization, resulting in polar Sm–Ge bonds. Notably, a pseudogap is observed at about −0.35 eV. Integration of the DOS from −12 eV up to the pseudogap yields 27.7 electrons per formula unit (*e*/f.u.), which is very close to the 28 valence electrons expected for a barrelane-like Ge_5_^8–^ cluster. Consequently, 1.3 *e*/f.u. are found in the region between the pseudogap and *E*_F_, suggesting a formal scenario analogous to that proposed for Eu_3_Ge_5_, but comprising one excess electron per formula unit: (Sm^3+^)_3_[(2*b*)­Ge^2–^]_3_[(3*b*)­Ge^–^]_2_ × 1*e* ^–^.

Calculated QTAIM effective charges (*Q* ^eff^) of +1.06 for Sm1, +1.05 for Sm2, and −0.60, −0.63, and −0.65 for Ge1, Ge2, and Ge3, respectively, are consistent with the electronegativity difference of the constituting atoms. The significantly lower *Q* ^eff^(Sm) compared to the formal +3 value is a typical feature observed in both binary^63–65^ and ternary^66–69^ tetrelides, which has been associated with the presence of covalent *RE*–Ge interactions.

To give insight into the chemical bonding, a topological analysis of ELI-D is performed. Probably due to the open-core–shell treatment of the 4*f* states of Sm, several discontinuities in the ELI-D were obtained, hindering its reliable analysis. This effect can likely be attributed to the pair-volume function rather than to the electron density, which does not exhibit similar features, thus enabling the derivation of QTAIM charges.

Therefore, in order to gain an in-depth understanding of the chemical bonding in Sm_3_Ge_5_, given the Sm trivalent nature, La_3_Ge_5_ was selected as a model compound. This was simulated by simply replacing Sm with La while keeping all structural parameters unchanged. To validate this approach, several tests were necessary. First, both the electronic structures, represented by the DOS, and the effective charges must not show significant differences. Second, the topology of the ELI-D, as well as the entire bonding analysis, must be compared with related compounds to ensure that the results are not affected by the applied computational approach. To this aim, two phases were selected: the experimentally determined La_3_Sn_5_ compound (see Section 8 in the Supporting Information); and the hypothetical La_3_Ge_5_ compound obtained by full geometry optimization (see Section 9 in the Supporting Information).

The DOS curves (Figure 6) do not show significant differences. The effective charges obtained for the model La_3_Ge_5_ compound (+1.13 for La1, +1.21 for La2, −0.67 for Ge1, −0.71 for Ge2, and −0.73 for Ge3) are analogous to those observed for Sm_3_Ge_5_. The rare-earth elements exhibit nearly identical values (difference <0.1) and Ge atoms follow the same trend, i.e., |*Q* ^eff^(Ge1)| < |*Q* ^eff^(Ge2)| < |*Q* ^eff^(Ge3)|. The overall charge transfer is slightly higher when Sm is substituted with La. Given these similarities, the bonding results obtained for the model compound La_3_Ge_5_ are presented below and are assumed to be reasonably transferable to Sm_3_Ge_5_.

The topology of ELI-D shows the presence of maxima (attractors) between Ge1–Ge3 and Ge2–Ge3, indicating covalent bonds. No attractors are found between Ge1–Ge2 along two edges of the Ge_5_ pyramid nor between the intercluster Ge2–Ge2 contacts (see Figure 7, to the right). It is important to keep in mind that these are the longest Ge–Ge distances, being >2.80 Å, both in the experimental and in the optimized structure.

**Figure 7:**
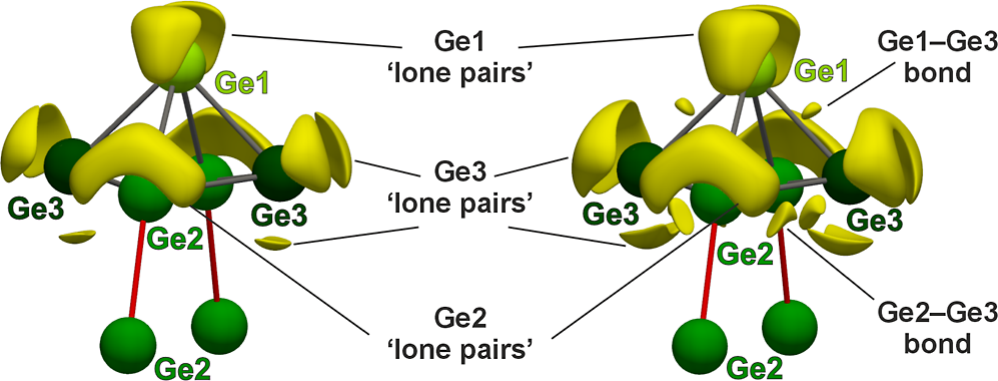
ELI-D distribution around the Ge_5_ square pyramids displayed by means of isosurfaces for the values of 1.200 (left) and 1.175 (right). Gray and red sticks indicate endohedral and exohedral contacts, respectively.

This scenario reveals relevant analogies with the ELF topology obtained for both Yb_3_Ge_5_ and Eu_3_Ge_5_, supporting a [1.1.1]­barrelane-like cluster, namely, a bicyclo[1.1.1]­pentagermanide anion, obtained by removing 8H^+^ from the hypothetic Ge_5_H_8_ molecule. This is in agreement with the previously described formal picture comprising (2*b*) and (3*b*)Ge species and one excess electron. The attractors related to the Ge–Ge homopolar bonds are located slightly off the edges of the square pyramid, likely due to angular strain associated with bond angles close to 60° within the Ge_5_ substructure, a situation previously reported for other highly strained clusters.^70,71^

Finally, it is interesting to highlight that the lack of ELI-D attractors along the shortest intercluster (exohedral) contacts, i.e., Ge2–Ge2, is consistent with the conclusions drawn for La_3_Sn_5_, which were primarily based on crystal orbital overlap populations (COOP).^4^ Indeed, the ELI-D for La_3_Sn_5_ obtained in this work (Figure S4) shows the same topology. The Integrated Crystal Orbital Hamilton Population (ICOHP) values related to Ge–Ge contacts obtained in Yb_3_Ge_5_, which features a lower number of valence electrons compared to Sm_3_Ge_5_ due to its average oxidation state of +2.4, decrease significantly with increasing interatomic distances, with the exohedral contact exhibiting the lowest value (0.68 eV/bond), which is approximately half of that of the two shortest Ge–Ge contacts.^34^ It is worth noting that in the earlier study, no computational attention was devoted to adequately address the electronic correlation of the partially filled 4*f* states of trivalent ytterbium. From a position-space perspective, further insight on the eventual interactions along the exohedral Ge2–Ge2 and intracluster (endohedral) Ge1–Ge2 contacts may be gained through delocalization indices (DI) and the ELI-D (relative) Laplacian, which enables the visualization of bonding interactions also in those cases where competing factors prevent the appearance of ELI-D maxima.^72^ Such a kind of investigation will be the object of future investigations. However, the absence of exohedral ELI-D attractors supports the trend reported in Figure 5, displaying that the Ge2–Ge2 exohedral distances are mainly influenced by size effects, in contrast to the endohedral distances (Figure S2), which remain largely unchanged regardless of the *M* metal involved. This suggests that endohedral interactions play a key role in stabilizing the crystal structure.

Focusing on Ge lone pairs, the only Ge species showing the expected classical scenario is (2*b*)­Ge1, with two lone pair-like localization domains per Ge atom (see Figure 8). On the contrary, Ge2 and Ge3 display one and three lone pair-like ELI-D attractors, respectively. Up to this point, the term “lone pair-like” is preferred over “lone pair”, as a definitive interpretation requires deeper insight into the bonding scenario, particularly between Ge and the rare-earth atoms, which is provided here through the ELI-D/QTAIM basins intersection. Obtained valence ELI-D basins are visualized in Figure 8 and the obtained position-space bonding parameters are listed in Table 3.

**Figure 8:**
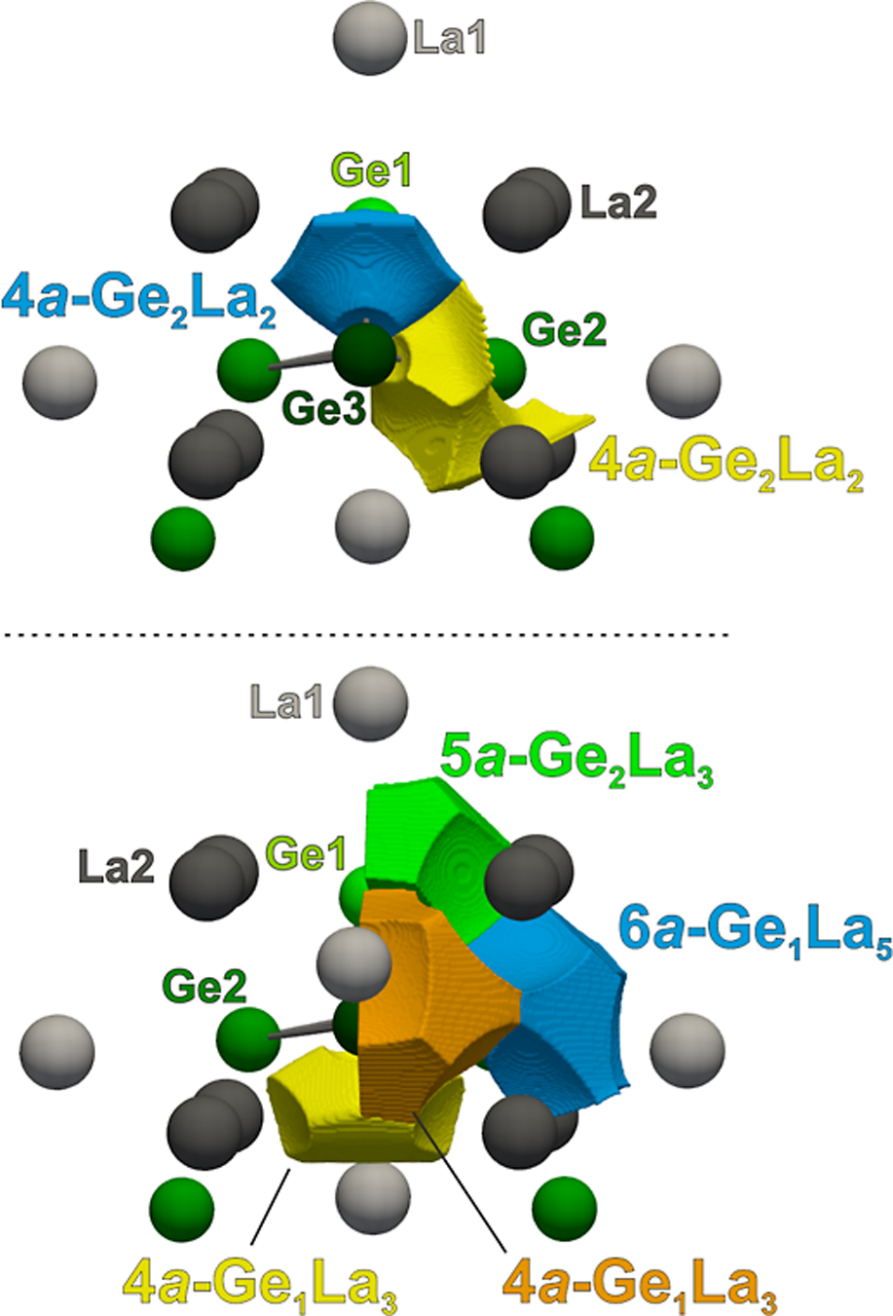
Shapes and atomicity of the ELI-D bond (top) and lone pair-like (bottom) basins of La_3_Ge_5_, displayed around a Ge_5_ square pyramidal cluster surrounded by neighboring La atoms.

**Table 3:** Bonding Parameters for La_3_Ge_5_ from the Position-Space Analysis^a^

ELI-D basin (*B*_i_)	color in Figure 8	atomicity Ge_n_La_h_	$\mathrm{N̅}\left(B_{i}\right)$	$\sum_{j=1}^{n}p\left(B_{i}^{{Ge}_{j}}\right)$	$\sum_{j=1}^{h}p\left(B_{i}^{{La}_{j}}\right)$
Ge1–Ge3	blue (top)	Ge_2_La_2_	1.15	0.98	0.02
Ge2–Ge3	yellow (top)	Ge_2_La_2_	1.53	0.95	0.05
lpGe1	green (bottom)	Ge_2_La_3_	2.10	0.91	0.09
lpGe2	blue (bottom)	Ge_1_La_5_	3.13	0.89	0.11
lpGe3	orange (bottom)	Ge_1_La_3_	1.51	0.89	0.11
lpGe3	yellow (bottom)	Ge_1_La_3_	1.15	0.91	0.09

a The atomicity, average electronic population $\mathrm{N̅}\left(B_{i}\right)$ , and bond fraction are listed for each ELI-D valence basin (*B*_*i*_). Label “lp” indicates lone pair-like basins.

The Ge1–Ge3 bond basin is intersected by one Ge1, one Ge3, and two La2 QTAIM atoms, leading to an atomicity of four, 4*a* (see Figure 8 and Table 3). However, due to the tiny contribution of La species to the bonding population, with a total bond fraction of 0.02, also corresponding to 0.02 electrons (*e* ^–^), this basin can be interpreted as effectively two-atomic (2*a*-Ge_2_), with 1.13 *e* ^–^ almost equally contributed by the germanium atoms, corresponding to a homopolar interaction. The typical underpopulation of bonding basins and the corresponding overpopulation of lone pair basins, relative to the ideal value of two electrons, is observed. Nevertheless, it is interesting to highlight that the Ge2–Ge3 bond is more populated than the Ge1–Ge3 (1.53 vs 1.15 *e* ^–^), revealing a trend opposite to that of interatomic distances (2.71 vs 2.66 Å), suggesting a difference in the nature of these two interactions, as confirmed by the results of the ELI-D/QTAIM intersection. In fact, the basin population of 1.53 *e* ^–^ is not equally contributed by the two germanium atoms, with bond fractions of 0.70 and 0.25 for Ge2 and Ge3, respectively (the sum being 0.95, as reported in Table 3), corresponding to 1.07 and 0.39 *e* ^–^ (see the scheme reported in Figure S3). This result indicates polar character for the covalent Ge2–Ge3 bond. The remaining 0.07 *e* ^–^ are contributed by two La species, resulting in a total bond fraction of 0.05 (Table 3); these values, although small, are higher than those found for the Ge1–Ge3 bonds, providing further evidence of the greater complexity of the bonding scenario revealed by position-space analysis compared to the formal one.

Focusing on the lone pair-like ELI-D basins, they all can be interpreted as multiatomic polar bonds, with total bond fractions for lanthanum of 0.09 or 0.11, emphasizing the importance of heteroatomic interactions, in agreement with previously published results for La_3_Sn_5_.^4^ The 5*a*-Ge_2_La_3_ basin deserves additional attention as it comprises two germanium QTAIM atoms in its atomicity, an unusual feature for this class of compounds. As expected, only Ge1 significantly contributes to the bond population with 1.84 *e* ^–^, corresponding to a bond fraction of 0.88, while Ge2 contributes 0.06 *e* ^–^. However, this finding, together with the shape of this basin, which shares a surface with the core basin of Ge2 (Figure S8), may be considered as an indication of endohedral interactions between Ge1 and Ge2. Finally, it is important to highlight that some of the aforementioned bonding features are not specific to La_3_Ge_5_, as revealed by additional calculations performed for La_3_Sn_5_. These include the contribution of Sn2 to the Sn1 lone pair-like basin (*N̅*(lpSn1) = 2.10 *e* ^–^; *p*(lpSn1^Sn2^) = 0.06, corresponding to 0.12 e^–^) and the polarity of the Sn2–Sn3 bonds (*N̅*(Sn2–Sn3) = 1.35 e^–^; *p*(Sn2–Sn3^Sn2^) = 0.68; *p*(Sn2–Sn3^Sn3^) = 0.25, resulting from contributions of 0.92 and 0.34 e^–^, respectively; see Table S9). At this point, it is particularly interesting to note that, although the optimized unit cell volume of the hypothetical La_3_Ge_5_ is larger than that of Sm_3_Ge_5_, consistent with both the high-pressure synthesis of the latter and the larger atomic radius of La, the bonding scenario resulting from the position-space analysis remains essentially unchanged. The optimized crystal structure of La_3_Ge_5_ is in agreement with the data in Table S5 and Figure S2, showing increased intercluster distances while the intracluster ones remain nearly constant, further supporting the observed bonding similarities. These results therefore suggest that the structure can tolerate analogous bonding scenarios as long as the intracluster distances within the Ge_5_ units are preserved, being largely unaffected by variations in the intercluster separation.

Differential scanning calorimetry measurements of the samples (Figure 9) show two distinct signals upon heating, one with an onset temperature of 475(10) K and another with an onset of 530(10) K plus a tiny shoulder pointing toward two overlapping effects. In samples heated to 520 K (above the first effect), (*oF*64)­Sm_3_Ge_5_ is identified by powder X-ray diffraction. A second sample is heated to a temperature of 630 K (above the second effect), which gives rise to additional diffraction lines of (*h* *P*16)­Sm_3_Ge_5_ and an unknown side phase. The transition behavior is consistent with (*oS*32)­Sm_3_Ge_5_ being a metastable high-pressure phase.

**Figure 9:**
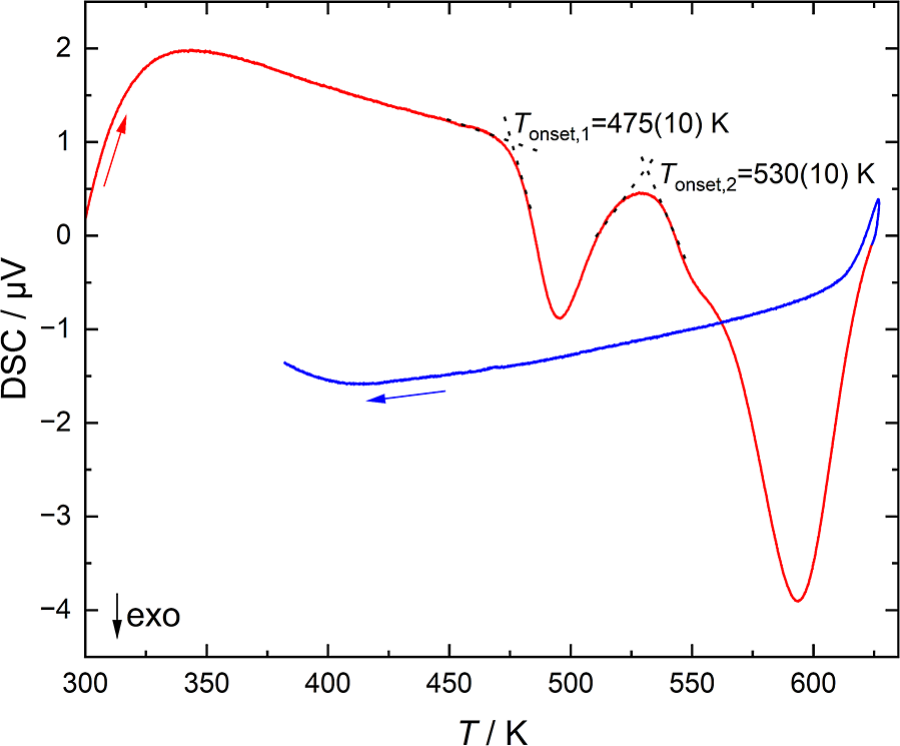
DSC measurement of Sm_3_Ge_5_ taken upon heating (red curve) and cooling (blue curve) in the range of 300–630 K with a heating rate of 10 K min^–1^ at ambient pressure.

Magnetization measurements (see Figure S9) indicate the magnetic ordering of the main phase starting at 21.5 K. Due to the low ordered moment, we suppose that the ordered spin structure is basically antiferromagnetic but that it has a weak ferromagnetic component, i.e., from spin canting. A broad ferromagnetic signal around 102 K is assigned to the magnetic ordering of a side phase as it is not associated with a significant thermal effect (see below). A drastic decrease in magnetization is seen when the isotherms in the magnetically ordered range are compared with those in the paramagnetic range (Figure S10).

For temperatures above about 150 K, susceptibility *χ* = *M*/*H* is field-independent. The temperature dependence of the paramagnetic susceptibility of samarium does not follow a Curie–Weiss type law but is known to exhibit van-Vleck behavior.^73,74^ Indeed, we observe a shallow minimum of *χ*(*T*) at approximately 330 K, which is typical for the van-Vleck paramagnetism of the 4*f* ^5^ configuration of Sm^3+^ (the corresponding maximum of the 1/*χ*(*T*) data is shown in the inset of Figure S9). Here, the low-lying states of the *J* = 7/2 multiplet with higher angular momentum start to get thermally populated besides the higher-energy crystal field (CF) levels of the ground-state multiplet ^6^ *H*_5/2_.^75^

The specific-heat capacity data for Sm_3_Ge_5_ (Figure 10) reveal two effects coinciding with the temperatures of the magnetic phase transitions, a larger one with a maximum at 20.4 K and a second smaller signal at 14.5 K (Figure 10). Interestingly, the latter transition is not visible in the magnetization data. Its magnetic signature is probably too weak to be visible, because of the background caused by the ferromagnetic component. Measurements in high magnetic fields reveal a weak gradual increase of the upper transition temperature, while the lower-temperature transition does not shift at all (see Figure S11). Such a weak field dependence of magnetic phase boundaries is frequently observed for Sm^3+^ compounds due to the small Landé *g*-factor of the ion. At temperatures around 102 K, there is no obvious anomaly of *C*_p_(*T*). This corroborates the interpretation of the ferromagnetic signal at this temperature as originating from a small amount of side phase with ferromagnetic order.

**Figure 10:**
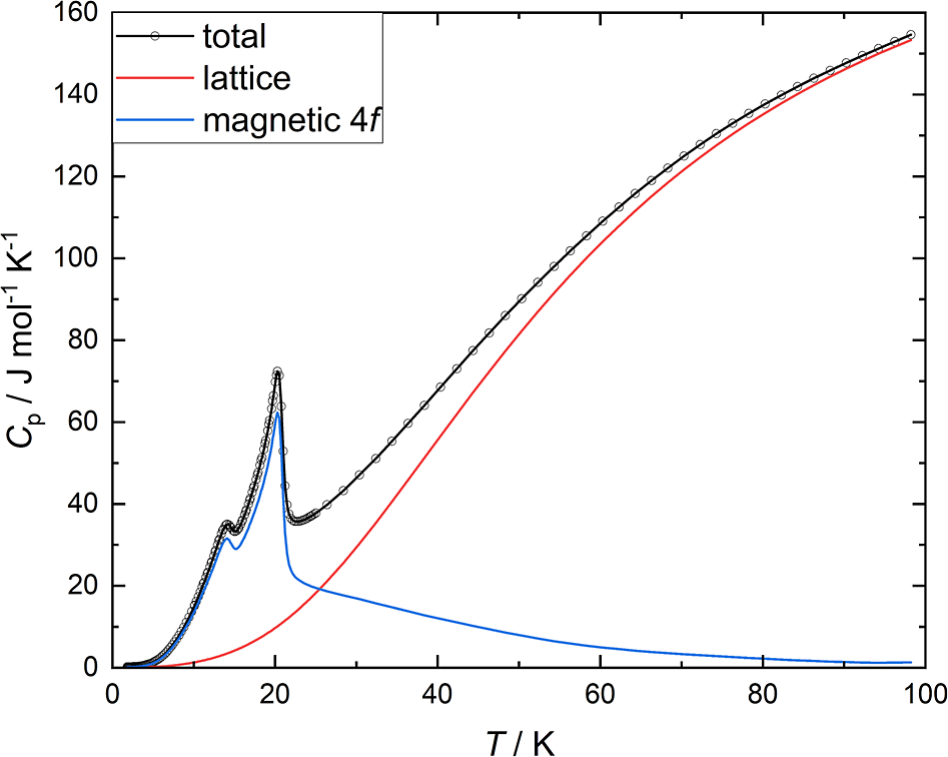
Specific heat capacity of Sm_3_Ge_5_. The red and blue lines show the lattice and 4*f*-electron magnetic contributions, respectively.

In order to substantiate the electron configuration of the 4*f* states of Sm and to validate the assigned oxidation state of the ion, we now estimate the magnetic contribution to the heat capacity *C*_magnetic_(*T*) and analyze the magnetic entropy *S*_magnetic_(*T*). To that end, a conjectural lattice heat capacity curve, *C*_lattice_(*T*), is calculated using the Debye lattice model.^76^ Adopting a Debye temperature of 235 K for 8 atoms, *C*_lattice_(*T*) (Figure 10, red curve) smoothly joins the experimental *C*_p_(*T*) curve for *T* > 100 K. The magnetic contribution, *C*_magnetic_ = *C*_p_ – *C*_lattice_, from the magnetic ordering peaks and the Schottky anomaly (from thermal excitations into higher CF states) is shown in Figure 10 by the blue curve.

Integration of *C*_magnetic_/*T* up to 100 K results in *S*_magnetic_ = 1.76 *R* per Sm atom. This value matches well with *R* ln 6 ≈ 1.8 *R* for the full thermal excitation of the ^6^ *H*_5/2_ ground-state multiplet of the 4*f* ^5^ configuration of Sm^3+^. Further, *S*_magnetic_(*T*) just above the magnetic ordering peak is around *R* ln 4, which is compatible with the 3 Sm ions in Sm_3_Ge_5_ having quasi-quartet ground states. However, there are two Sm crystallographic sites in Sm_3_Ge_5_, and therefore, conclusions on the degeneracy of the CF ground states are not unique.

The nature of the second transition at 14.5 K remains ambiguous as there are two plausible scenarios (Figures 10 and S11). In one, both Sm sites order at 20.4 K, and then the second transition may be interpreted as a spin-reordering from an intermediate- into a low-temperature spin structure. Alternatively, the large 20.4 K transition peak may be due to the ordering of the Sm2 (8*e*) sublattice, while the smaller peak at 14.5 K represents the antiferromagnetic ordering of the Sm1 (4*c*) species.

The electrical resistance *R*(*T*) (Figure S12) measured on a polycrystalline sample is very high, which is probably due to microcracks as well as some germanium-rich phase at the grain boundaries. We therefore abstained from calculating resistivity from these data; however, the temperature dependence of *R*(T) suggests the metallic conductivity of the main phase.

In conclusion, high-pressure, high-temperature synthesis paved the way to hp-Sm_3_Ge_5_ crystallizing in a Pu_3_Pd_5_-type structure, making it the first germanide of this structure type in which the metal atoms adopt the oxidation state +3. Thermal analysis suggests that the compound is a high-pressure phase, which is metastable at ambient conditions. The compound shows metallic conductivity, and magnetization and heat capacity measurements reveal two magnetic phase transitions in-line with DFT calculations. The topology of the calculated Electron Localizability Indicator (ELI-D) supports the interpretation of the Ge_5_ units as strained bicyclo[1.1.1]­pentagermanide clusters composed of two- and three-bonded germanium species. This leads to a formal description analogous to that proposed for Eu_3_Ge_5_ but featuring one excess electron per formula unit due to the coherent trivalent nature of the lanthanide. Moreover, in-depth analysis reveals the presence of multiatomic bonds between germanium and the rare-earth species, polar covalent interactions among the germanium atoms at the base of the distorted Ge_5_ pyramidal clusters, and evidence of subsidiary endohedral Ge–Ge interactions (Ge1–Ge2), indicating a complex overall bonding picture that defies simple rationalization based on formal charge transfer considerations.

## Supplementary Material





## Notes

Video visualizing the ELI-D distribution (MP4)
Additional experimental details, materials, and methods, including information on synthesis, microstructure analysis, crystal structure refinement, comparison of interatomic distances with related compounds, chemical bonding calculations, and physical property measurements (PDF)
