# Supplementary material for: Ge5 Clusters in the Trivalent Rare-Earth Compound Sm3Ge5
Source: Inorg Chem. 2025 Sep 13;64(38):19217–26. doi: 10.1021/acs.inorgchem.5c02417 (PMC12486209; doi:10.1021/acs.inorgchem.5c02417)
Supplement: Supplementary file 2 [file ic5c02417_si_002.pdf]

SUPPORTING INFORMATION

Ge_5 Clusters in the Trivalent Rare-Earth Compound Sm_3Ge_5

Julia-Maria Hübner,[1*] Riccardo Freccero,[2] Wilder Carrillo-Cabrera,[3] Marcus Schmidt,[3] Walter Schnelle,[3] and Ulrich Schwarz[3]

[1] Faculty of Chemistry and Food Chemistry, TUD Dresden University of Technology, 01062 Dresden, Germany

[2] Dipartimento di Chimica e Chimica Industriale, Università degli Studi di Genova, Via Dodecaneso 31, I-16146 Genova, Italy

[3] Max Planck Institute for Chemical Physics of Solids, 01187 Dresden, Germany

E-mail: julia-maria.huebner@tu-dresden.de

1. Synthesis
2. Analysis of microstructure
3. Crystal structure refinement
4. Comparison of interatomic distances in selected cluster compounds M_3Tt_5 (M = Sr, Ba, La, Sm, Eu; Tt = Ge, Sn)
5. Structure model of the antiferromagnetic Sm_3Ge_5 phase used in the DFT simulations.
6. Crystal structure data obtained from quantum chemical optimization
7. Details on the Ge2–Ge3 bond forming the base of the Ge_5 pyramidal clusters
8. Electronic structure and position space bonding results for La_3Sn_5.
9. Electronic structure and position space bonding results in fully optimized La_3Ge_5.
10. Details of the lone pair-like ELI-D basin of Ge1
11. Physical properties measurements

1. Synthesis

Table S1. Results of PXRD analysis of selected samples. As precursor mixtures were arc melted prior to high-pressure, high-temperature synthesis, a Sm excess was used to account for evaporation loss.

**Composition Precursor**	**Synthesis Conditions**	**PXRD Results**
Sm_3Ge_5, +6 % Sm	7 GPa, 1150°C- 30 min, 550°C-50 h*	$SmGe_{2-x}$ ($ThSi_2$-type)
Sm_3Ge_5, +6 % Sm	9.5 GPa, 1150°C- 300 min	Sm_3Ge_5 (Pu_3Pd_5-type), unknown phase
$SmGe_2$, +8 % Sm	9.5 GPa, 1150°C- 30 min, 550°C-300 min*	Sm_3Ge_5 (Pu_3Pd_5-type), $SmGe_3$
Sm_3Ge_5, +6 % Sm	9.5 GPa, 1150°C- 30 min, 550°C-300 min*	Sm_3Ge_5 (Pu_3Pd_5-type), unknown phase
Sm_3Ge_5, +9 % Sm	9.5 GPa, 950°C- 30 min, 700°C-300 min, cooling within 300 min*	Sm_3Ge_5 (Pu_3Pd_5-type), unknown phase
Sm_3Ge_5, +9 % Sm	9.5 GPa, 950°C- 60 min, 600°C-300 min, cooling within 300 min*	Sm_3Ge_5 (Pu_3Pd_5-type), unknown phase
Sm_3Ge_5, +6 % Sm	9.5 GPa,1300°C-30 min, 550°C-10 h, cooling within 20h*	Sm_3Ge_5 (Pu_3Pd_5-type), unknown phase
Sm_3Ge_5, +6 % Sm	9.5 GPa, 1150°C- 30 min, 550°C-50 h*	Sm_3Ge_5 (Pu_3Pd_5-type), Sm_5Ge_3, $SmGe_3$
Sm_3Ge_5, +7.5 % Sm	11 GPa, 1000°C-60 min	SmGe, unknown phase, Sm_3Ge_5 (Pu_3Pd_5-type)
Sm_3Ge_5, +6 % Sm	12 GPa, 850°C-60 min	$SmGe_{2-x}$ ($ThSi_2$-type), unknown phase

*Long annealing and slow cooling programs were applied to facilitate crystal growth. However, no specimen suitable for single crystal diffraction could be obtained.

2. Analysis of microstructure

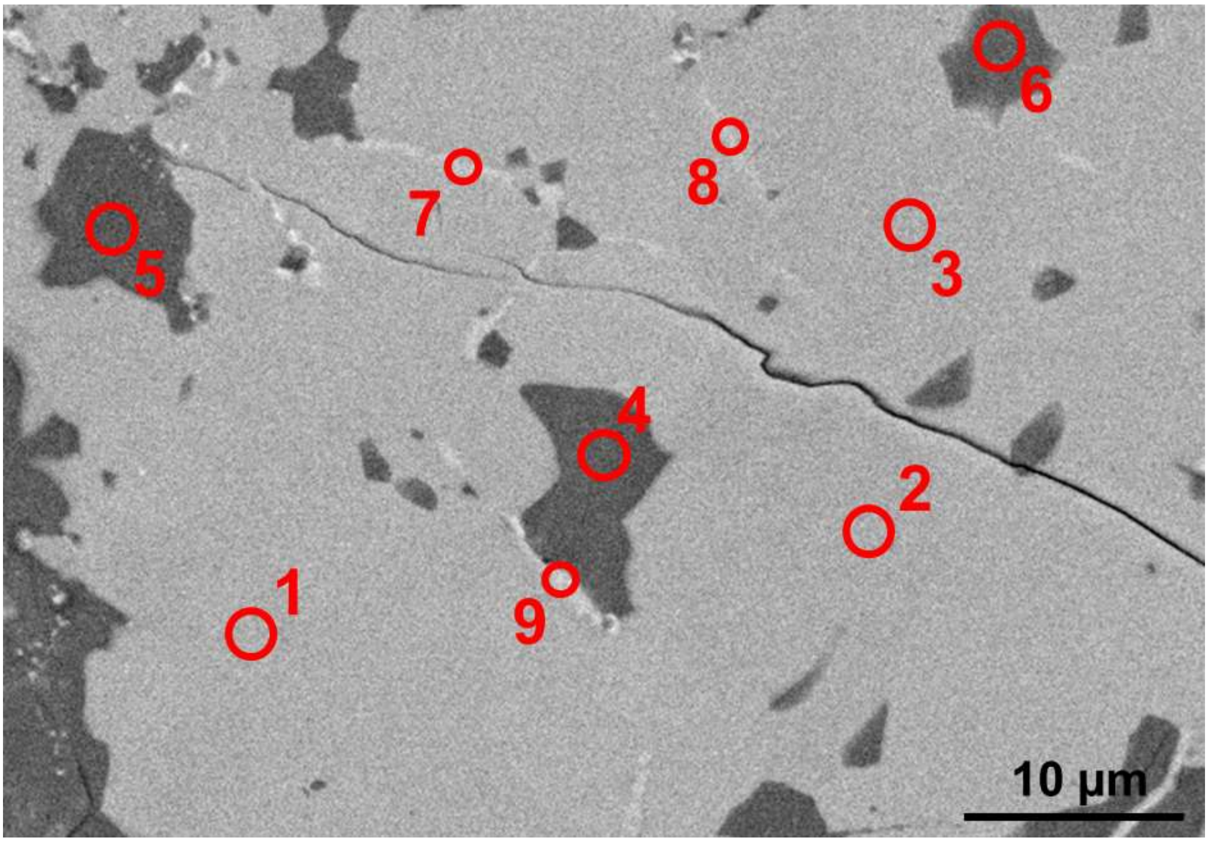

Figure S1. Scanning electron microscopy image of the same sample, as used for Rietveld refinement (Figure 1).

Table S2. Results of SEM/EDXS analysis in at.-%. Points correspond to positions marked in Figure S1.

Point ID	Sm	Ge
1	37.64	62.36
2	37.67	62.63
3	37.90	62.10
4	25.82	74.18
5	24.92	75.08
6	26.22	73.78
7	65.99	34.01
8	55.93	44.07
9	70.50	29.50

3. Crystal structure refinement

Table S3. Wyckoff positions, relative atomic coordinates, and displacement parameters U_{iso} for Sm_3Ge_5.

Atom	Site	*x*/*a*	*y*/*b*	*z*/*c*	U_{iso} / Å^2
Sm1	4*c*	0	0.6530(2)	¼	0.0122(5)
Sm2	8*e*	0.20018(9)	0	0	0.0114(3)
Ge1	4*c*	0	0.0426(4)	¼	0.0169(9)
Ge2	8*f*	0	0.3269(3)	0.4478(2)	0.0120(7)
Ge3	8*g*	0.2036(2)	0.2848(2)	¼	0.0191(7)

Table S4. Interatomic distances in Sm_3Ge_5.

Atom		Distance / Å	Atom		Distance / Å
Sm1	4· Sm2	3.8954(8)	Ge1	2· Ge2	2.878(3)
	4· Sm2	4.037(1)		2· Ge3	2.653(3)
	1· Ge1	2.947(3)		1· Sm1	2.947(3)
	2· Ge2	2.926(2)		4· Sm2	3.0840(6)
	2· Ge2	3.121(2)	Ge2	1· Ge1	2.878(3)
	2· Ge3	2.967(2)		1· Ge2	2.806(3)
	2· Ge3	3.382(2)		2· Ge3	2.729(2)
Sm2	2· Sm1	3.8954(8)		1· Sm1	2.926(2)
	2· Sm1	4.037(1)		1· Sm1	3.121(2)
	1· Sm2	3.775(1)		2· Sm2	3.151(2)
	2· Sm2	3.8964(3)		2· Sm2	3.156(1)
	2· Ge1	3.0840(6)	Ge3	1· Ge1	2.653(3)
	2· Ge2	3.151(2)		2· Ge2	2.729(2)
	2· Ge2	3.156(1)		1· Sm1	2.967(2)
	2· Ge3	3.052(1)		1· Sm1	3.382(2)
	2· Ge3	3.238(1)		2· Sm2	3.052(1)
				2· Sm2	3.238(1)

4. Comparison of distances

Table S5. Dihedral angles and interatomic distances (inner-pyramidal d_{in} and inter-pyramidal d_{int}) of Tt_5 square pyramidal clusters of selected Pu_3Pd_5-type tetrel compounds. Additionally, the average distance d_{in} (weighted by distance count) was scaled by the d_{int} to account for differences in the sizes of the involved atoms.

Compound	Dihedr. angle / °	d_{in} / Å	d_{int} / Å	$d_{in-Ø}$ / d_{int}	Ref.
Ba_3Ge_5	187.0	2.5934 to 2.7052	3.817	0.69	5
Eu_3Ge_5	190.8	2.620 to 2.753	3.291	0.81	21
Sm_3Ge_5	199.0	2.653(3) to 2.878(3)	2.806(3)	0.97	this work
Yb_3Ge_5	192.1	2.634 to 2.787	2.957	0.91	34
$Ba_3Ge_{2.8}Sn_{2.2}$	191.6	2.772 to 2.943	3.617	0.79	3
$Ca_{0.96}Sr_{2.04}Sn_5$	191.0	3.002 to 3.164	3.343	0.91	S1
Sr_3Sn_5	190.4	2.992 to 3.153	3.397	0.90	3
Ba_3Sn_5	190.2	2.992 to 3.146	3.695	0.82	3
La_3Sn_5	195.4	3.034 to 3.234	3.166	0.98	4
Yb_3Sn_5	189.8	3.028 to 3.156	3.307	0.93	35
Ba_3Pb_5	189.4	3.164 to 3.289	3.739	0.85	3

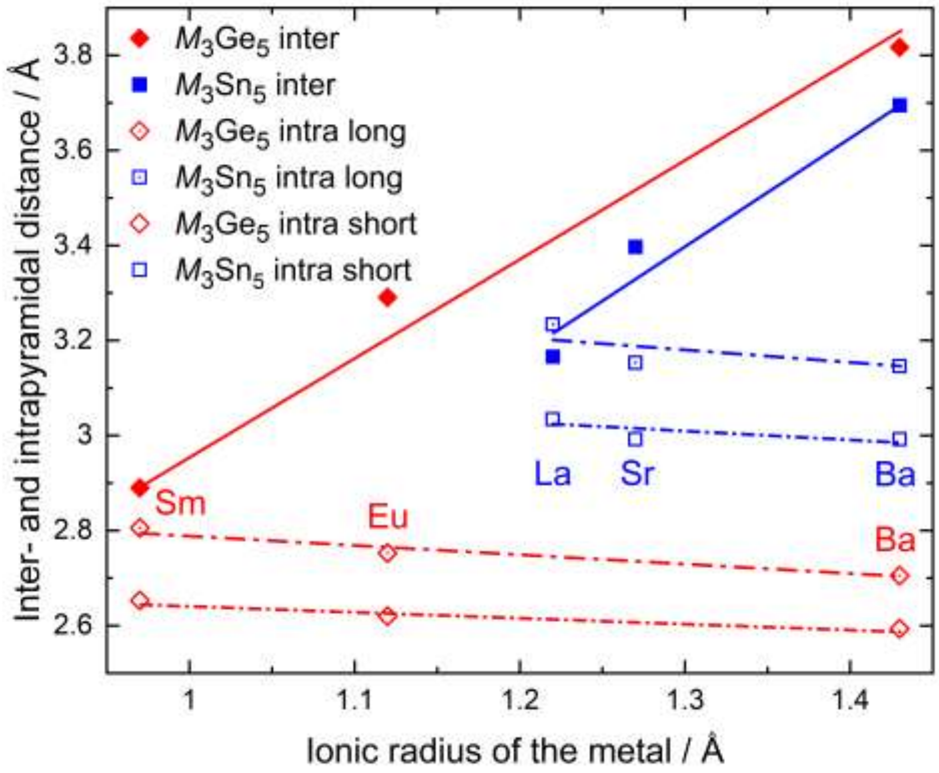

Figure S2. Inter- and intra-pyramidal distances in dependence of the atomic radius of the cation. Straight lines are guides to the eye.

5. Structure model of the antiferromagnetic Sm_3Ge_5 phase used in the DFT simulations.

Table S6. Antiferromagnetic model of Sm_3Ge_5 used as the input structure for DFT calculations (space group *Pmma*, *a* = 9.67056, *b* = 9.42813, *c* = 7.56296 Å). The optimized atomic coordinates are reported in Table S7, in the *Cmcm* space group.

Atom	Site	*x*/*a*	*y*/*b*	*z*/*c*
Sm1 (up)	*2e*	¼	0	0.6496
Sm2 (up)	4*h*	0	0.69842	½
Sm3 (down)	*2f*	¼	½	0.1496
Sm4 (down)	4*g*	0	0.1984	0
Ge1	2*e*	¼	0	0.0457
Ge2	2*f*	¼	½	0.5457
Ge3	4*i*	0.4437	0	0.3231
Ge4	4*j*	0.4437	½	0.8231
Ge5	4*k*	¼	0.2062	0.2900
Ge6	4*k*	¼	0.7062	0.7900

6. Crystal structure data obtained from quantum chemical optimization

Table S7. Atomic coordinates of Sm_3Ge_5 obtained from quantum chemical optimization using the experimentally refined lattice parameters.

Atom	Site	*x*/*a*	*y*/*b*	*z*/*c*
Sm1	4*c*	0	0.64777	¼
Sm2	8*e*	0.2001	0	0
Ge1	4*c*	0	0.03952	¼
Ge2	8*f*	0	0.32753	0.44633
Ge3	8*g*	0.20272	0.28505	¼

7. Details on the Ge2–Ge3 bond at the base of the Ge_5 pyramidal clusters

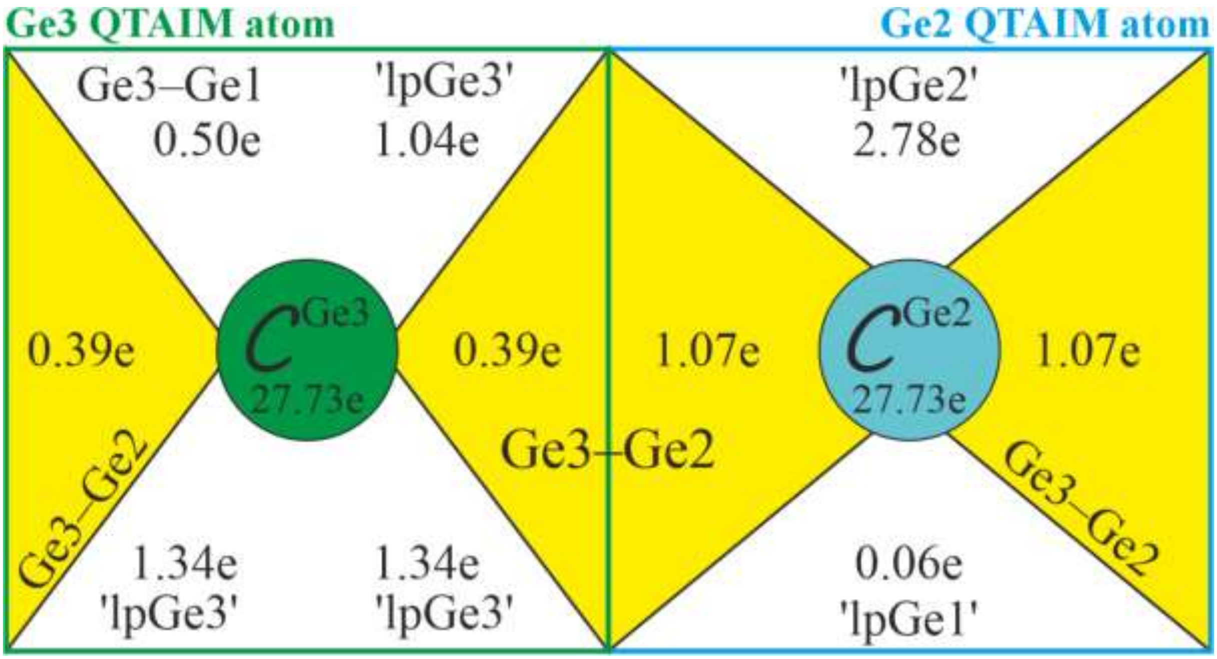

Figure S3. Schematic representation of the decomposition of the populations of Ge3 and Ge2 QTAIM atoms, represented by green (left) and blue (right) rectangles, into ELI-D basins contributions. The yellow parts correspond to the Ge2–Ge3 ELI-D basins (it is indicated with the same colour as in Figure 8 (top) in the main text). The 0.39 and 1.07 e results from the integration of the ED within the regions of the Ge2–Ge3 basins intersected by the Ge3 and Ge2 atoms, respectively ($\bar{N}(Ge2 - Ge3^{Ge3}) = 0.39$; $\bar{N}(Ge2 - Ge3^{Ge2}) = 1.07$). The name of the other ELI-D basins (valence and core) are reported together with their contribution to the QTAIM populations.

The QTAIM atomic population can be obtained by summing the number of valence and core electrons obtained by integrating the ED within ELI-D valence and core basins, respectively:

$$\bar{N}(X) = \bar{N}_{val}^{ELI}(X) + \bar{N}(C^X)$$

Atomic population of Ge3 derived from ELI-D contributions (FigureS3 to the left):

$$\bar{N}(Ge3) = (0.39 \cdot 2) + (1.34 \cdot 2) + 0.50 + 1.04 + 27.73 = 32.73$$

$$Q^{eff}(Ge3) = Z(Ge) - \bar{N}(Ge3) = 32 - 32.73 = -0.73$$

Atomic population of Ge2 derived from ELI-D contributions (Figure to the right):

$$\bar{N}(Ge2) = (1.07 \cdot 2) + 0.06 + 2.78 + 27.73 = 32.71$$

$$Q^{eff}(Ge2) = Z(Ge) - \bar{N}(Ge2) = 32 - 32.71 = -0.71$$

8. Electronic structure and position space bonding results for La_3Sn_5

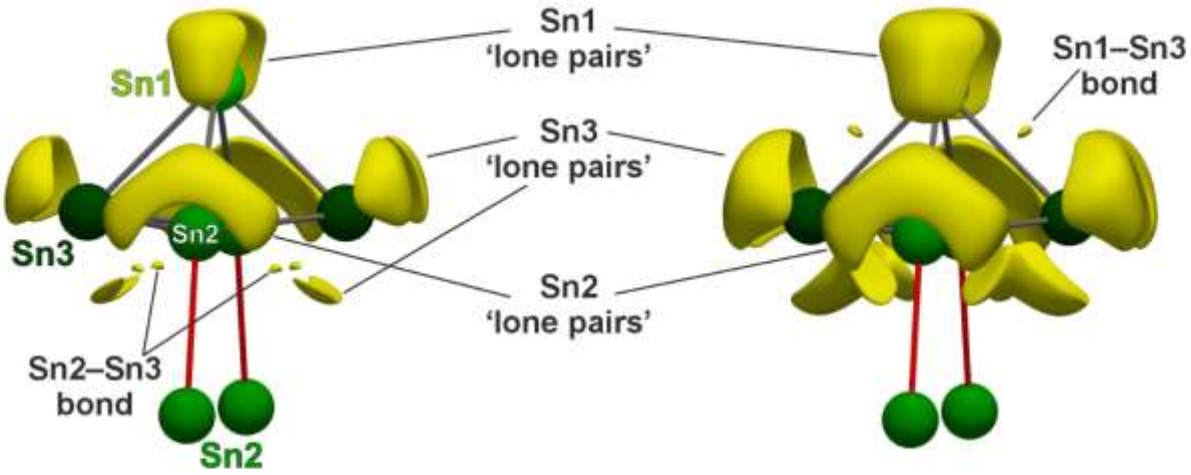

Figure S4. ELI-D distribution around the Sn_5 square pyramids displayed by means of isosurfaces for the values of 1.130 (left) and 1.095 (right). Grey and red sticks indicate endohedral and exohedral contacts, respectively. The localization domains corresponding to Sn3 lone pairs, pointing towards the La1 species located underneath the square base of the Sn_5 pyramid, enclose two bifurcated attractors.

Table S8. QTAIM effective charges.

**La1**	**La2**	**Sn1**	**Sn2**	**Sn3**
+1.09	+1.15	–0.61	–0.68	–0.71

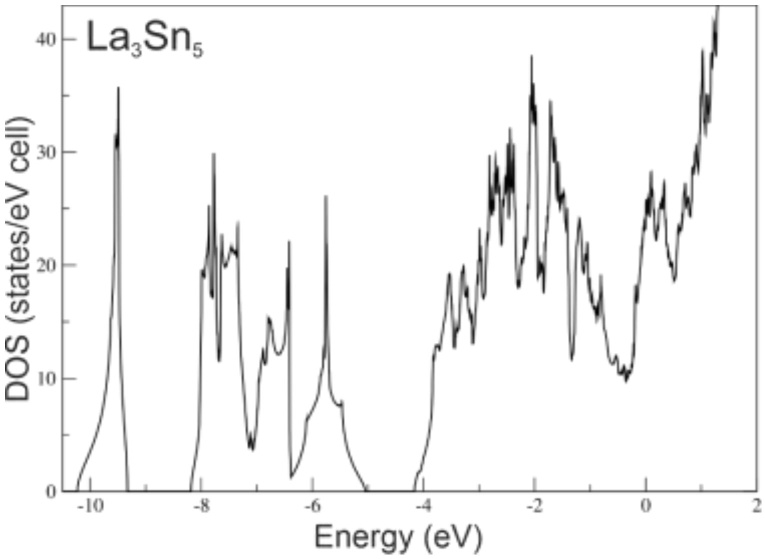

Figure S5. Total electronic DOS for La_3Sn_5.

Table S9. Bonding parameters for La_3Sn_5 from the position-space analysis.

ELI-D basin (B_i)	*Atomicity* Sn_nLa_h	$\bar{N}(B_i)$	$\sum_{j=1}^{n} p(B_i^{\mathrm{Sn}_j})$	$\sum_{j=1}^{h} p(B_i^{\mathrm{La}_j})$
Sn1-Sn3	Sn_2La_2	1.10	0.97	0.02
Sn2-Sn3	Sn_2La_2	1.35	0.93	0.05
lpSn1	Sn_2La_3	2.10	0.91	0.09
lpSn2	Sn_1La_5	3.19	0.88	0.12
lpSn3	Sn_1La_3	1.50	0.88	0.12
lpSn3	Sn_1La_3	1.19	0.90	0.10

9. Electronic structure and position space bonding results in fully optimized La_3Ge_5

Full crystal structure optimization (atomic positions and unit cell parameters) was performed by means of the all-electron FHI-aims code [S2] at the DFT/PBE level of theory. The predefined default "tight" basis sets were selected for each atomic species. The Brillouin zone was sampled with a 10x12x10 *k*-point mesh; a Gaussian broadening function with a default width of 0.01 eV was used and scalar-relativistic effects for all electrons were included within the ZORA approximation.

For consistency with the other results, the position space bonding analysis was performed based on FPLO/DGrid calculations conducted starting from the optimized structure. The same computational setup described in the main text was employed.

Table S10. Crystal structure of the hypothetical La_3Ge_5 obtained after full geometry optimization. (space group *Cmcm*, *a* = 9.8025, *b* = 7.9322, *c* = 9.8722 Å).

Atom	Site	*x*/*a*	*y*/*b*	*z*/*c*
La1	4*c*	0	0.64974	¼
La2	8*e*	0.20492	0	0
Ge1	4*c*	0	0.04711	¼
Ge2	8*f*	0	0.31401	0.05732
Ge3	8*g*	0.19562	0.28618	¼

Table S11. QTAIM effective charges.

La1	La2	Ge1	Ge2	Ge3
+1.23	+1.29	–0.71	–0.76	–0.79

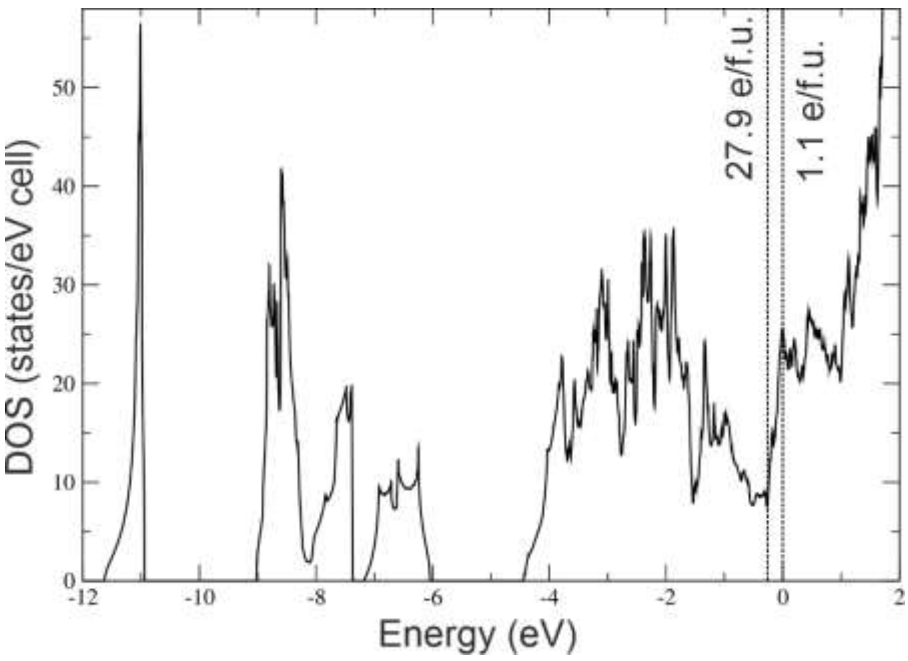

Figure S6. Total electronic DOS for the hypothetical La_3Ge_5 after geometry optimization. The same integrals shown in the main text are reported.

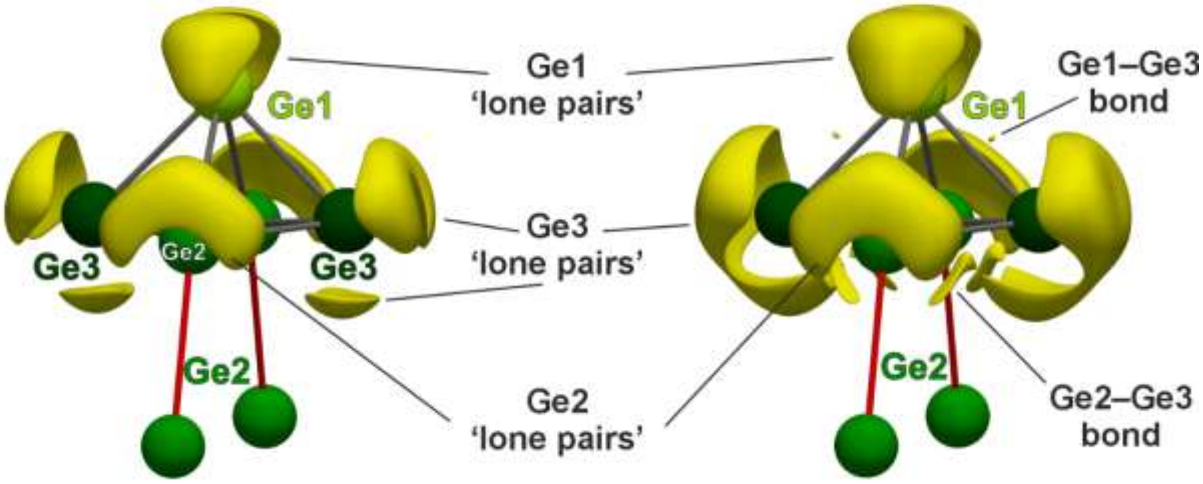

Figure S7. ELI-D distribution around the Ge_5 square pyramids displayed by means of isosurfaces for the values of 1.200 (left) and 1.712 (right). Grey and red sticks indicate endohedral and exohedral contacts, respectively. The localization domains corresponding to the Ge2–Ge3 bonds enclose two bifurcated attractors.

Table S12. Bonding parameters for the fully optimized, hypothetical La_3Ge_5 compound derived from the position-space analysis.

ELI-D *basin (B_i)*	*Atomicity* Ge_nLa_h	$\bar{N}(B_i)$	$\sum_{j=1}^{n} p(B_i^{Ge_j})$	$\sum_{j=1}^{h} p(B_i^{La_j})$
Ge1-Ge3	Ge_2La_2	1.02	0.98	0.01
Ge2-Ge3	Ge_2La_2	1.37	0.96	0.04
lpGe1	Ge_2La_3	2.20	0.92	0.08
lpGe2	Ge_1La_5	3.28	0.89	0.11
lpGe3	Ge_1La_3	1.50	0.89	0.11
lpGe3	Ge_1La_3	1.39	0.91	0.09

Also in this case:

1) Ge2 contributes to the Ge1 lone pair-like basin ($\bar{N}$(lpGe1) = 2.20 e⁻; $p(\mathrm{lpGe1}^{Ge2}) = 0.05$ and $p(\mathrm{lpGe1}^{Ge1}) = 0.87$corresponding to 0.12 e⁻ and 1.91 e⁻, respectively).
2) Ge2–Ge3 bond is polar: $\bar{N}$(Ge2–Ge3) = 1.37 e⁻; $p(\mathrm{Ge2-Ge3}^{Ge2}) = 071$; $p(\mathrm{Ge2-Ge3}^{Ge3}) = 0.25$, resulting from contributions of 0.98 and 0.34 e⁻, respectively.

10. Details of the lone pair-like ELI-D basin of Ge1

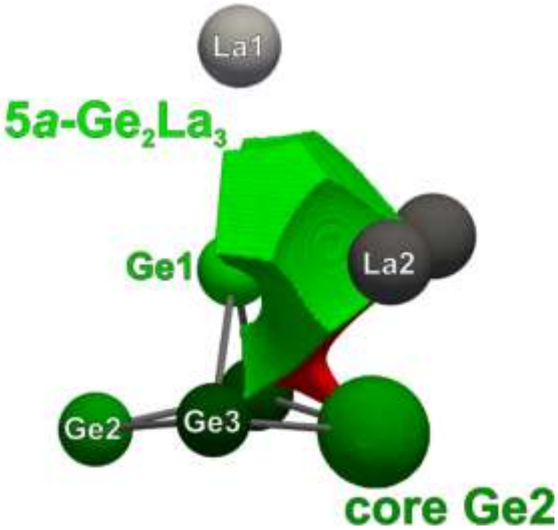

Figure S8. Lone pair-like ELI-D basin of the Ge1 species (light green), corresponding to a polar covalent five atomic 5*a*-Ge_2La_3 bond ($Ge1_1Ge2_1La2_2La1_1$), together with the core basin of Ge2 (dark green). The red part corresponds to the portion of the 5*a*-Ge_2La_3 basin intersected by the Ge2 QTAIM atom.

11. Physical properties measurements

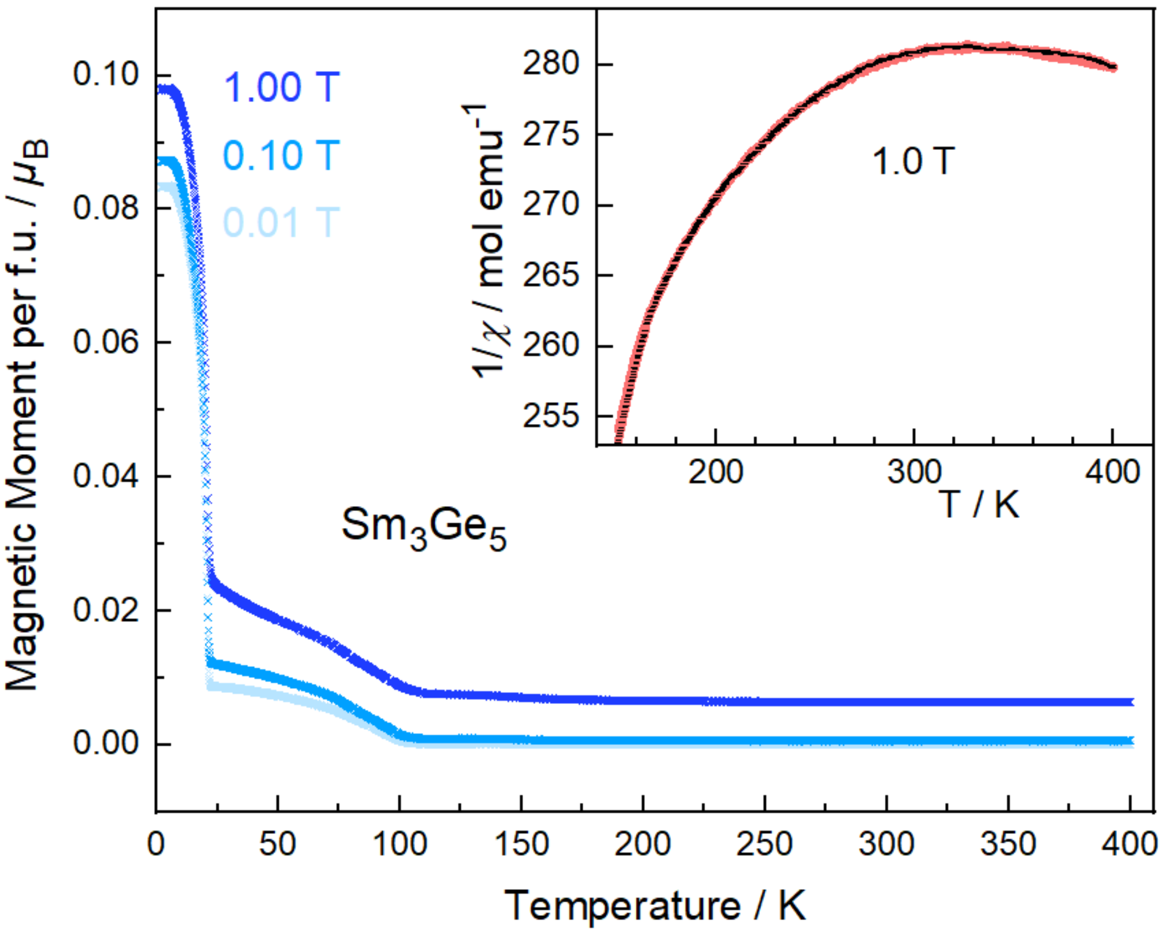

Figure S9. Temperature dependence of the magnetic moment of the Sm_3Ge_5 sample measured in different external fields. The insert shows inverse magnetic susceptibility in the paramagnetic regime of the main phase. The orange curve corresponds to data measured upon warming after zero-field cooling. The field cooling data shown in blue coincide almost perfectly.

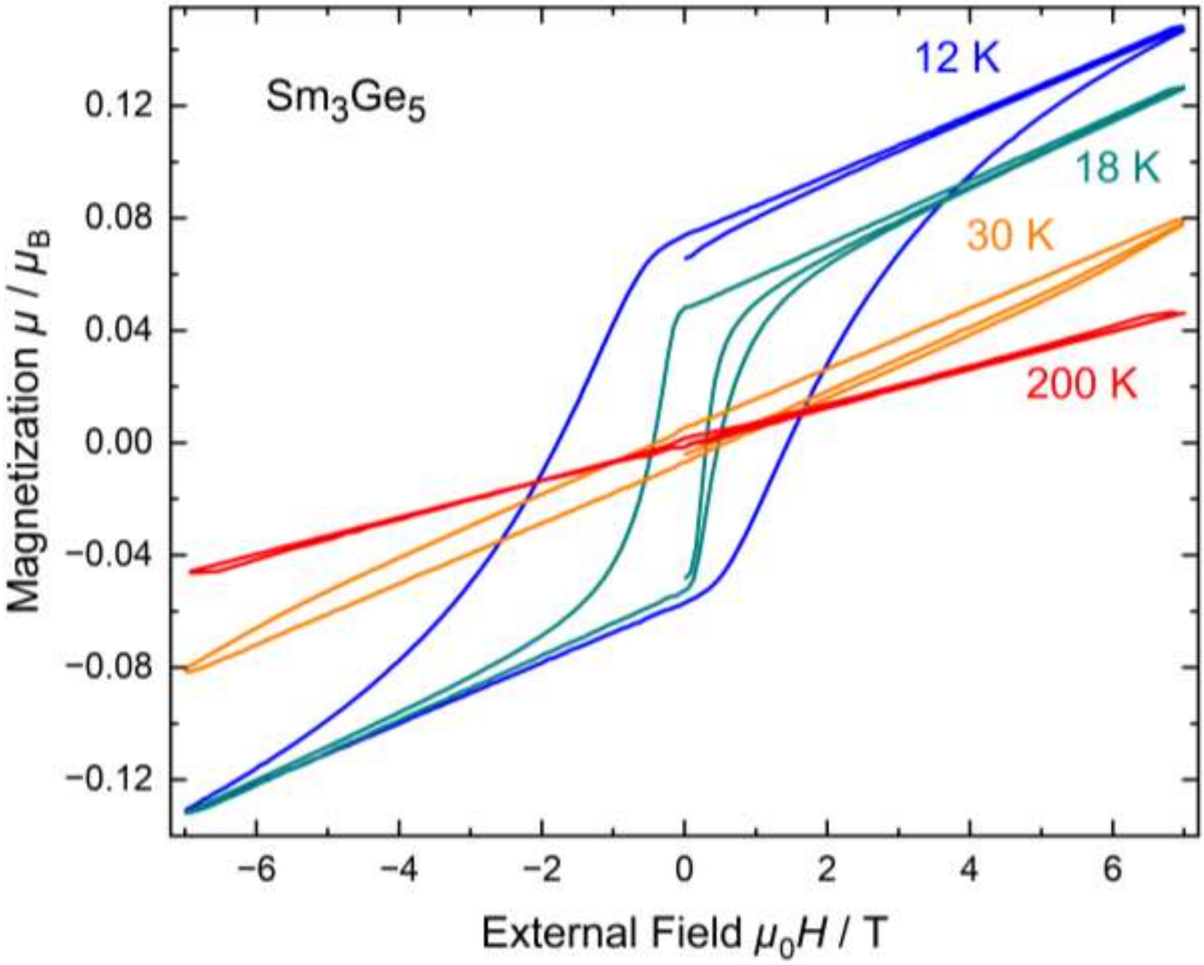

Figure S10. Isothermal magnetization curves at selected temperatures below (12 K, 18 K) and above (30 K, 200 K) the magnetic ordering of the main phase.

A drastic decrease in magnetization is seen when comparing the isotherms in the magnetically ordered range with those in the paramagnetic range. The small remaining magnetization signal with pronounced hysteresis in the paramagnetic range (of the main phase) is assigned to the ferromagnetic order of a side phase below approximately 102 K.

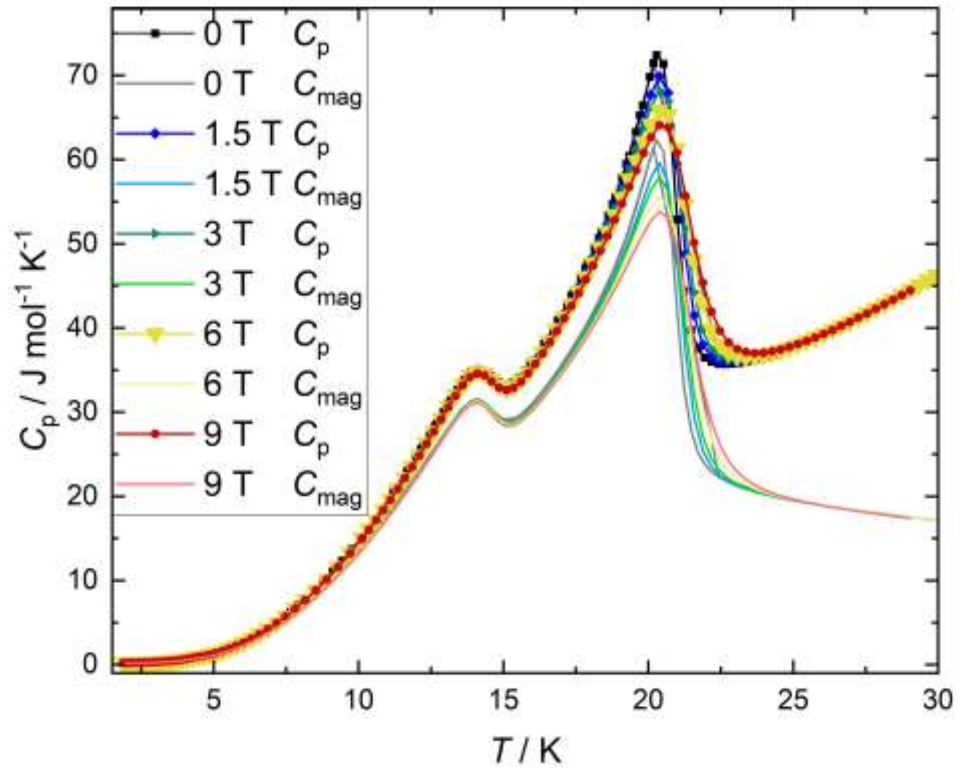

Figure S11. Magnetic field dependence of the specific heat C_p with magnetic heat capacity $C_{mag} = C_p - C_{lattice}$ ($C_{lattice}$ as calculated from Debye model with θ_D = 235 K for 8 atoms.

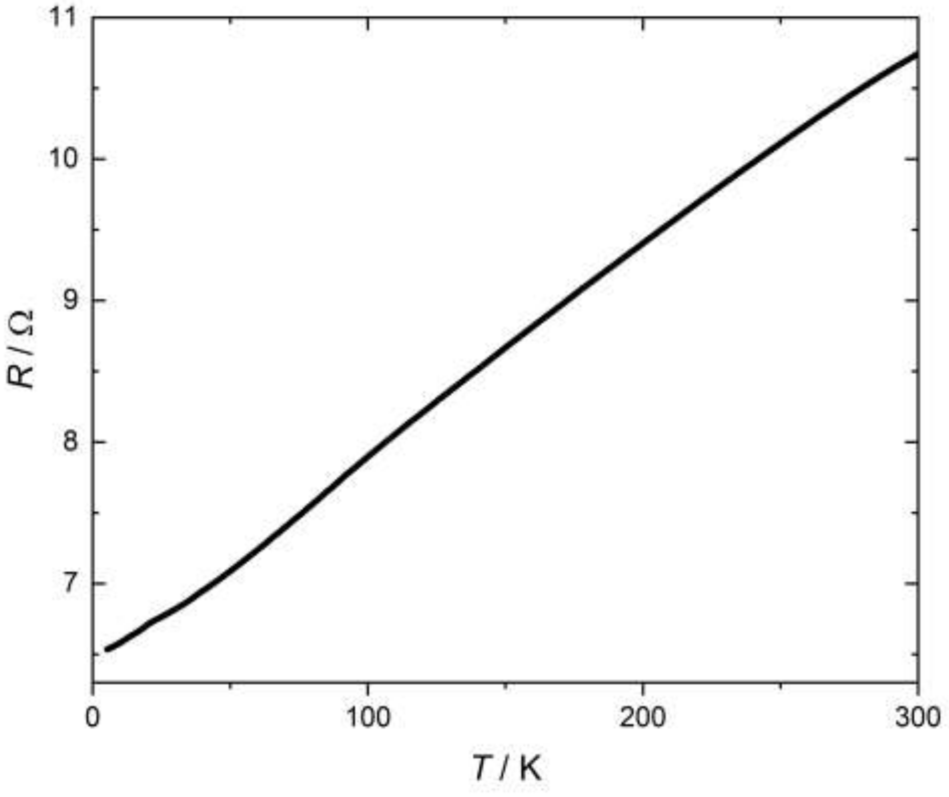

Figure S12. Electrical resistance of the Sm_3Ge_5 sample in dependence of temperature.

Additional References (only used in the Supporting Information)

[S1] Gupta, S.; Ganguli, A.K. Evidence of incipient bond-stretching isomerism in $Sr_{2.04(1)}Ca_{0.96(1)}Sn_5$ from variable-temperature structural studies. *Inorg. Chem.* **2005**, *44*, 7443–7448.

[S2] Blum, V.; Gehrke, R.; Hanke, F.; Havu, P.; Havu, V.; Ren, X.; Reuter, K.; Scheffler, M. Ab initio molecular simulations with numeric atom-centered orbitals, *Comput. Phys. Commun*. **2009**, 180, 2175-2196.
